# Step-growth titanium-catalysed dehydropolymerisation of amine–boranes†
†Electronic supplementary information (ESI) available. See DOI: 10.1039/c7sc05395a


**DOI:** 10.1039/c7sc05395a

**Published:** 2018-03-06

**Authors:** Titel Jurca, Theresa Dellermann, Naomi E. Stubbs, Diego A. Resendiz-Lara, George R. Whittell, Ian Manners

**Affiliations:** a School of Chemistry , University of Bristol , Cantock's Close , Bristol BS8 1TS , UK . Email: Ian.Manners@bristol.ac.uk

## Abstract

Precatalysts active for the dehydropolymerisation of primary amine–boranes are generally based on mid or late transition metal. We have found that the activity of the precatalyst system formed from Cp^R^_2_TiCl_2_ and 2*n*BuLi towards the dehydrogenation of the secondary amine–borane Me_2_NH·BH_3_, to yield the cyclic diborazane [Me_2_N–BH_2_]_2_, increases dramatically with increasing electron-donating character of the cyclopentadienyl rings (Cp^R^). Application of the most active precatalyst system (Cp^R^ = η-C_5_Me_5_) to the primary amine–borane MeNH_2_·BH_3_ enabled the first synthesis of high molar mass poly(*N*-methylaminoborane), [MeNH–BH_2_]_*n*_, the BN analogue of polypropylene, by an early transition metal such as catalyst. Significantly, unlike other dehydropolymerization precatalysts for MeNH_2_·BH_3_ such as [Ir(POCOP)H_2_], skeletal nickel, and [Rh(COD)Cl]_2_, the Ti precatalyst system was also active towards a range of substrates including BzNH_2_·BH_3_ (Bz = benzyl) yielding high molar mass polymer. Moreover, in contrast to the late transition metal catalysed dehydropolymerisation of MeNH_2_·BH_3_ and also the Ziegler–Natta polymerisation of olefins, studies indicate that the Ti-catalyzed dehydropolymerization reactions proceed by a step-growth rather than a chain-growth mechanism.

## Abstract 2

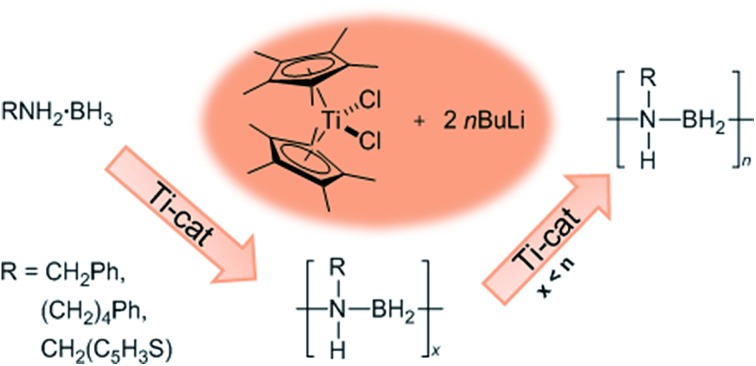
Titanium-catalysed dehydropolymerisation of primary amine–boranes was found to proceed *via* a step-growth rather than a chain-growth mechanism.

## Introduction

Catalysis plays a pivotal role in molecular and macromolecular C–C bond forming chemistry. The development of comparable reactions for the catenation of other p-block elements, however, has proceeded at a markedly slower pace. Nonetheless, the identification of useful target main group molecules and materials over the past decade has prompted significant progress in the field.^1^ For example, catalytic dehydrocoupling/dehydrogenation of amine–boranes has become an area of widespread interest, largely motivated by potential applications in hydrogen storage^2^ and transfer,^3^ and the formation of novel ceramic thin films and polymeric materials.^1^,^4^ The latter can be regarded as BN analogues of polyolefins, but with distinct properties and possible applications, for example as piezoelectrics and precursors to boron-based solid state materials.^4^ Consequently, a wide variety of catalyst systems have been developed to promote the dehydrogenation of amine–boranes in general, with the vast majority based on mid to late transition metals (*e.g.* Re,^5^ Fe,^6^ Ru,^7^ Rh,4e,^8^ Ir4c,^9^ and Ni^10^).^11^ With regards to the dehydropolymerisation of primary amine–boranes using Brookhart's catalyst, [IrH_2_(POCOP)] (POCOP = 2,6-bis(di-*tert*-butylphosphinito)benzene),^12^ our group has reported the formation of high molar mass (*M*_n_ > 50 000 g mol^–1^) [MeNH–BH_2_]_n_ (**5**) from MeNH_2_·BH_3_ (**4**).4a,c Other middle to late metal catalysts, such as [CpFe(CO)_2_]_2_,6*b* [Rh(Ph_2_P(CH_2_)_4_PPh_2_)]^+^,4*i* and [Rh(κ^2^-*P*,*P*-xantphos){η^2^-H_2_B(CH_2_CH_2_^*t*^Bu)·NMe_3_}]^+^,4e,8e have also been shown to be effective in this role, and in certain cases key mechanistic information has been elucidated. These polymerisations thus appear to proceed by a chain-growth coordination-insertion mechanism.1e,4c,e Metal-free routes involving free, transient aminoborane monomers have also been recently reported, but remain mechanistically unclear.4g,^13^

**Scheme 1:**
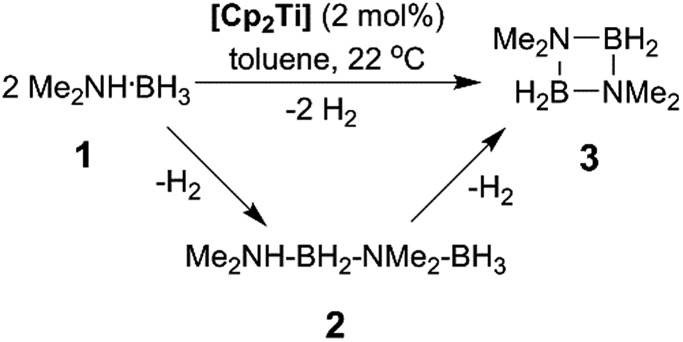
Titanocene-catalysed dehydrocoupling of Me_2_NH·BH_3_ (**1**) to give [Me_2_N–BH_2_]_2_ (**3**).

In addition to our report of [CpFe(CO)_2_]_2_ (ref. 6b) as an example of an earth abundant transition metal catalyst, we also described the use of the group 4 metallocene precatalysts Cp_2_TiCl_2_ (**6a***vide infra*)/two equiv. of *n*BuLi or Cp_2_Ti(PMe_3_)_2_ as reasonably efficient dehydrocoupling catalysts for the secondary amine–borane Me_2_NH·BH_3_ (**1**), yielding the cyclodiborazane [Me_2_N–BH_2_]_2_ (**3**) (Scheme 1).^14^ Others11b,^15^,^16^ have also reported the use of neutral Ti^II^ and Zr^II^, and also cationic Zr^IV^ precatalysts for the dehydrocoupling of **1**. From these studies, two general reaction mechanisms have been proposed. Compound **1** may react with the active catalyst to form Me_2_N=BH_2_ as the intermediate, which then dimerizes to afford **3** in an off-metal process,^15^,^17^ as shown for late transition metal catalyst systems.1c,2a,10b,^18^ Alternatively, **1** may be dehydrocoupled to form the linear diborazane Me_2_NH–BH_2_–NMe_2_–BH_3_ (**2**) as the intermediate, which then yields **3** in a subsequent on-metal, ring-closing dehydrogenation step and indicates a rather different mechanism.^14^,^19^ Our group has also reported the preparation of paramagnetic Ti^III^ species related to the catalytic reaction,^20^ and identified the Ti^III^–amido–borane complex [Cp_2_Ti(NMe_2_BH_3_)] (**6b**, *vide infra*) as being more active than either [**6a** + 2*n*BuLi], or Cp_2_Ti(PMe_3_)_2_ for the dehydrocoupling of **1** to give **3** (*via***2**).^21^ To date, however, the polymerisation of the primary amine–borane MeNH_2_·BH_3_ using a catalyst system based on an early transition metals such as Ti or Zr has not be observed.

Herein, we report structure-correlated kinetic studies of different titanium based precatalyst systems for the dehydrogenation of the secondary amine–borane Me_2_NH·BH_3_ (**1**), and based on these results, the first successful dehydropolymerisation of primary amine–boranes, yielding high molecular weight polyaminoboranes, that proceeds by a step-growth rather than a chain-growth mechanism.

## Results

### Dehydrogenation of *N*,*N*-dimethyl amine–borane

**Chart 1:**
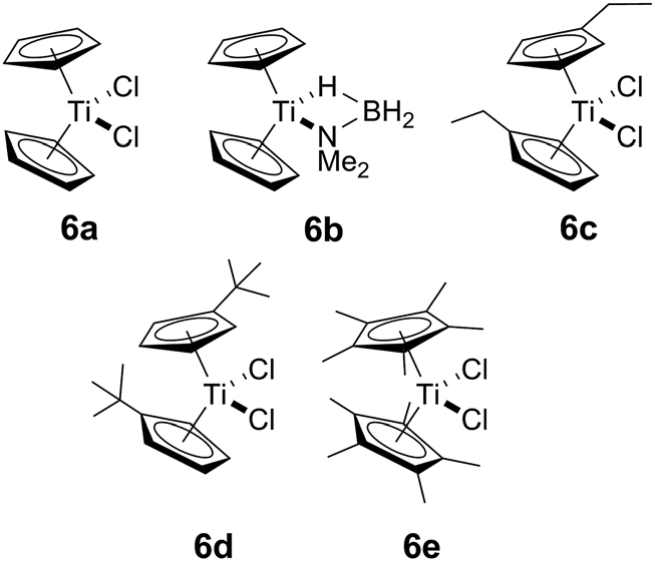
Ti-based amine–borane dehydrocoupling/dehydropolymerisation precatalyst components **6a–e**.

Our initial investigations were based on the influence of cyclopentadienyl ligand substitution on the activity of a series of two-component precatalysts, which were formed by Cp^R^_2_TiCl_2_ and 2*n*BuLi. We therefore explored the dehydrocoupling of amine–borane **1** (1 M in toluene) mediated by 2 mol% of [**6c–e** + 2*n*BuLi] at 22 °C in toluene. Previously reported precatalysts [**6a** + 2*n*BuLi]^11^ and Ti(iii) species **6b** (ref. 21) were also investigated under identical conditions for comparative purposes (Chart 1), as well as the reaction of [**6e** + 2*n*BuLi] in THF. All reactions were conducted in sealed J. Young NMR tubes, and monitored by ^11^B NMR spectroscopy.22*a* Rapid initial conversion of **1** (δ^11^B –13.8 ppm) to linear diborazane **2** (δ^11^B 1.6 ppm (internal BH_2_), –13.8 ppm (terminal BH_3_)) was detected, followed by slower subsequent conversion of **2** to cyclodiborazane **3** (δ^11^B 4.9 ppm), presumably with concomitant release of H_2_. The compounds (Me_2_N)_2_BH (δ^11^B 28.4 ppm) and Me_2_N=BH_2_ (δ^11^B 37.4 ppm) were also identified in the reaction mixture, but in very minor amounts (Fig. S1–S6†). All chemical shifts and coupling constants for the products were consistent with those reported in the literature.5b,^15^

**Fig. 1:**
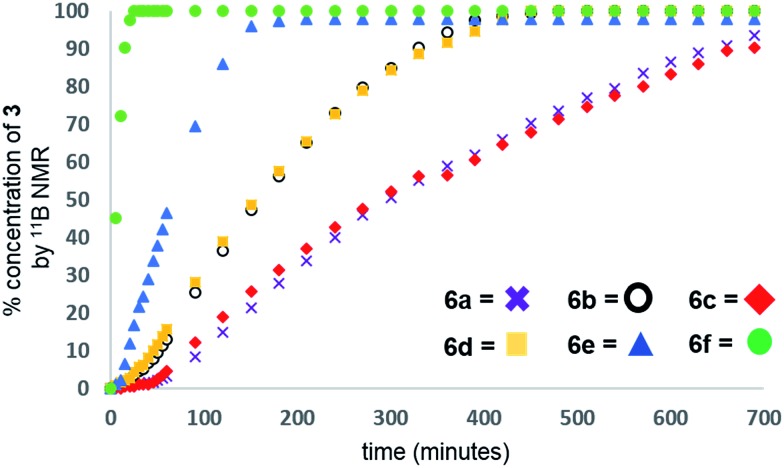
Reaction profiles for the formation of **3** from the catalytic dehydrocoupling of **1** with precatalysts [2 mol% **6a**,**c–f** + 2*n*BuLi], and **6b** as monitored by ^11^B{^1^H} (96 MHz, toluene-*d*_8_) NMR spectroscopy.

Precatalyst [**6a** + 2*n*BuLi] resulted in the slowest conversion to **3**, only reaching high (>90%) conversion after 690 min.22*b* Switching to precatalysts **6b**^20^ and [**6c** + 2*n*BuLi] resulted in an increased reaction rate, with reaction completion at 390 and 420 min, respectively. Most significantly, reactions with precatalysts [**6d**/**6e** + 2*n*BuLi] proceeded at a substantially faster rate, reaching complete conversion to **3** after 180 min for **6d**, and remarkably, in under 30 min in the case of **6e** (Fig. 1 and S6†). A change in solvent from toluene to THF for **6e** results in nearly no conversion of **1** after 12 h, despite the latter being a better solvent for **1**. This reduction in activity is therefore most probably caused by coordination of the solvent to the active site of the catalyst (Fig. S7†). The observed difference between **6d** and **6e** is particularly informative, as these ligands are effectively isosteric as indicated by the similar coordination gap aperture (cga) values of *ca.* 58 and 55°, respectively.^23^ In addition to influencing the rate and strength of substrate bonding, this feature would also be expected to similarly affect the existence of any off-cycle dimerization, or the formation of an “tucked-in complex”.^24^ They do, however, exhibit different electronic properties, as shown through IR spectroscopy of the corresponding [Cp^R^Fe(CO)_2_]_2_ complexes (*ν*(CO) for [Cp^R^Fe(CO)_2_]_2_ = 1762, 1938 and 1755, 1922 cm^–1^ for Cp^R^ = *t*BuC_5_H_4_ and C_5_Me_5_, respectively).^25^ This result strongly suggests that the trend of increasing reaction rate from **6a–e** is most probably a consequence of the increasing electron-donating character of the Cp^R^ ligands rather than any steric factor.

For the most active precatalyst [**6e** + 2*n*BuLi], this translated to a turnover frequency (TOF) of 141 h^–1^ (based on 45% conversion to **3** after 5 min, see Table S1†) and this value is in the range (35–420 h^–1^) reported for the conversion of **1** to **3** by isolable Cp^R^_2_Ti precatalysts.^15^ Increased reaction rates were also reported for these species on incorporation of electron donating SiMe_3_ groups on Cp^R^, however, the disambiguation of the role of steric and electronic effects was not possible. Nonetheless, dehydrocoupling with precatalysts [**6a**,**c–e** + 2*n*BuLi] and **6b** proceeded *via* linear diborazane **2** rather than Me_2_N=BH_2_ as the major intermediate, which differs from that reported for the isolable Ti^II^ precatalysts (Scheme 1).

### Dehydropolymerisation of primary amine–boranes

**Scheme 2:**
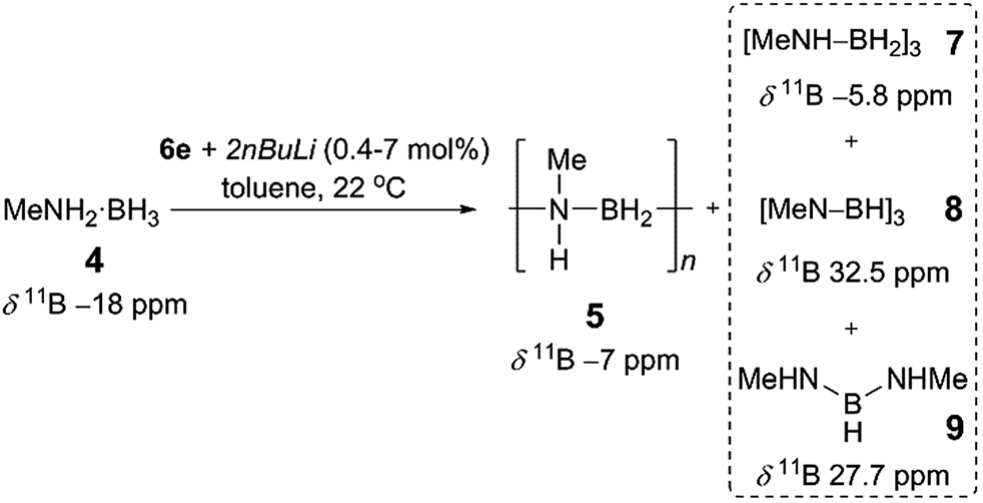
Catalytic dehydropolymerisation of **4** with precatalyst [**6e** + 2*n*BuLi] to give **5** and byproducts **7–9**.

Prompted by the high activity of precatalysts [**6d**/**6e** + 2*n*BuLi] towards **1** we endeavoured to test them towards the dehydropolymerisation of primary amine–borane **4**. Preliminary kinetic studies were conducted with *ca.* 2 mol% catalyst in toluene solution at 22 °C in sealed J. Young NMR tubes, and the reactions were monitored by ^11^B NMR spectroscopy (Fig. S8, S9†). For the reaction of [**6d** + 2*n*BuLi] with **4** the spectra show the instant formation of polyaminoborane **5** (δ^11^B –6.1 ppm, and –18 ppm assigned to the end-group) as well as the presence of (MeNH)_2_BH, **9** (δ^11^B 27.7 ppm), which formed presumably *via* redistribution of amine–borane **4**. Simultaneously, [MeNH–BH_2_]_3_, **7** (δ^11^B –5.8 ppm) could be detected, which was further dehydrogenated forming [MeN–BH]_3_**8** (δ^11^B 32.5 ppm) after *ca.* 5 h (product assignment based on the literature, see Fig. S8†).4a,c,6b Unreacted amine–borane **4** was still present in the reaction mixture even after 23 h. On the other hand [**6e** + 2*n*BuLi] led to a complete consumption of **4** after *ca.* 8 h and formation of predominantly polymer **5** and byproducts **7** (95% combined for **5** and **7**, as the peaks were unresolvable in the ^11^B NMR spectrum), **8** (4%) and **9** (minimal amounts) (Fig. S9 and S10†).^26^ It is noteworthy that in this case **7** and **8** are the only species observed after *ca.* 1 h. Based on these promising results we focused the remainder of our dehydropolymerisation studies on precatalyst [**6e** + 2*n*BuLi] (Scheme 2).

**Fig. 2:**
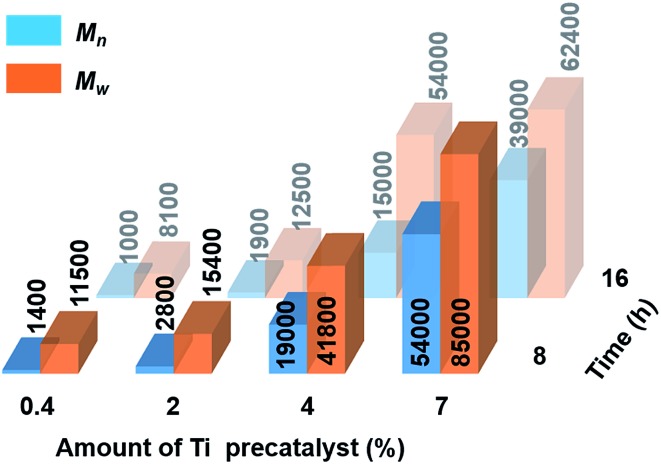
Graphical representation of molar mass (*M*_n_ and *M*_w_ in g mol^–1^) of **5** obtained from the reactions of **4** with precatalyst [**6e** + 2*n*BuLi] as a function of catalyst loading (0.4–7 mol%) and reaction time (8 h and 16 h) at 22 °C.

Catalytic dehydropolymerisation reactions of **4** were focused on the isolation and characterisation of polymer **5** with precatalyst [**6e** + 2*n*BuLi] and conducted in toluene (1.5 M in substrate) at 22 °C. To optimize the conditions for the formation of the high molecular weight polyaminoborane **5**, variable catalyst loadings from 0.4–7 mol% were screened initially at both 8 h and 16 h (see Scheme 2 and Fig. S11–S18†). After precipitation of the reaction mixture into cold hexanes and removal of both the soluble catalyst and byproducts, all reactions led to the isolation of white polymeric **5** (with yields of 53–72% limited by the above-mentioned side reactions), which was characterised by ^11^B NMR spectroscopy and Gel Permeation Chromatography (GPC). A steady increase in molar mass (*M*_n_) and a concomitant decrease in polydispersity index (PDI = *M*_w_/*M*_n_) was observed with increasing catalyst loading. Consistent with the former was the decreasing intensity of the end-group resonance (δ^11^B *ca.* –18 ppm) with respect to that of the main-chain (δ^11^B *ca.* –6 ppm) in the ^11^B NMR spectra (Fig. S11–S18†). This observation served to confirm the original assignment, and in combination with the absence of any well-resolved coupling in the corresponding proton-coupled spectra, suggests the lack of significant amounts of unreacted **4**. Increased reaction times (from 8 h to 16 h) only served to afford polyaminoboranes with lower *M*_n_ values (see Fig. 2, and Table S2†). The optimal conditions for the formation of **5** involved 7 mol% [**6e** + 2*n*BuLi] and 8 h reaction time, yielding polymer with *M*_n_ = 54 000 g mol^–1^ (PDI = 1.6).

**Scheme 3:**
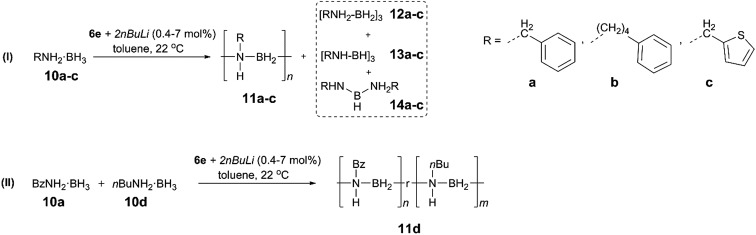
Catalytic dehydropolymerisation of **10a–c** (I) and of a mixture of **10a** and **10d** (II) with precatalyst [**6e** + 2*n*BuLi] to give polymers **11a–d** and the respective byproducts **12a–c**, **13a–c** and **14a–c**. The ratio of monomers **10a** and **10b** of the copolymer **11d** was determined by ^1^H NMR spectroscopy giving a n/m ratio of 2.

To extend the substrate scope of the dehydropolymerisation reaction, the *N*-benzyl (Bz) substituted amine–borane BzNH_2_·BH_3_ (**10a**) was reacted under previously optimised conditions, yielding a white, sparingly soluble precipitate (Scheme 3).^27^ GPC analysis of the THF-soluble fraction indicated the presence of high molar mass polymer **11a** with *M*_n_ = 101 700 g mol^–1^ (PDI = 1.15) (Fig. S28†). Further studies carried out on the dehydropolymerisation reaction showed the formation of byproducts **12a**, **13a** and **14a** after approximately 1 h, which is analogous to the results for the dehydropolymerisation of **4** (Fig. S20†). Surprisingly, previous attempts to dehydropolymerise this substrate using the well-established Ir catalyst [IrH_2_(POCOP)], skeletal nickel or [Rh(COD)Cl]_2_ have been unsuccessful and showed no reaction. These results encouraged us to perform similar dehydropolymerisation reactions using the *N*-4-phenylbutyl (**10b**) and the *N*-thiophenylmethyl amine–borane (**10c**) as substrates as well as an equimolar mixture of BzNH_2_·BH_3_ (**10a**) and *n*BuNH_2_·BH_3_ (**10d**) (Scheme 3). This yielded the homopolymers **11b** and **11c** and the copolymer **11d**, respectively. All reactions yielded high molar mass polymers with *M*_n_ = 349 100 g mol^–1^ (PDI = 1.30, **11b**), 95 600 g mol^–1^ (PDI = 1.29, **11c**) and 131 900 g mol^–1^ (PDI = 1.33, **11d**) (Table 1, Fig. S28 and S32†). In contrast to poly(*N*-benzylaminoborane) **11a**, the latter polymers (**11b–d**) were completely soluble and could be further characterized by ^1^H, ^13^C and ^11^B NMR spectroscopy and mass spectrometry (**11d**) (Fig. S21–S27, S29–S31 and S33†).

**Table 1:** Yields, molecular weights and polydispersity indices for isolated polymers **11a–c** from the reaction of **10a–c** with [**6e** + 2*n*BuLi] (7 mol%, 8 h, 22 °C)

	Yield (%)	Molecular weight *M*_n_ (g mol^–1^)	Molecular weight *M*_w_ (g mol^–1^)	PDI
**11a**	31	101 700	116 700	1.15
**11b**	61	349 100	453 700	1.30
**11c**	44	95 600	124 400	1.29
**11d**	44	131 900	175 400	1.33

### Mechanistic studies

**Fig. 3:**
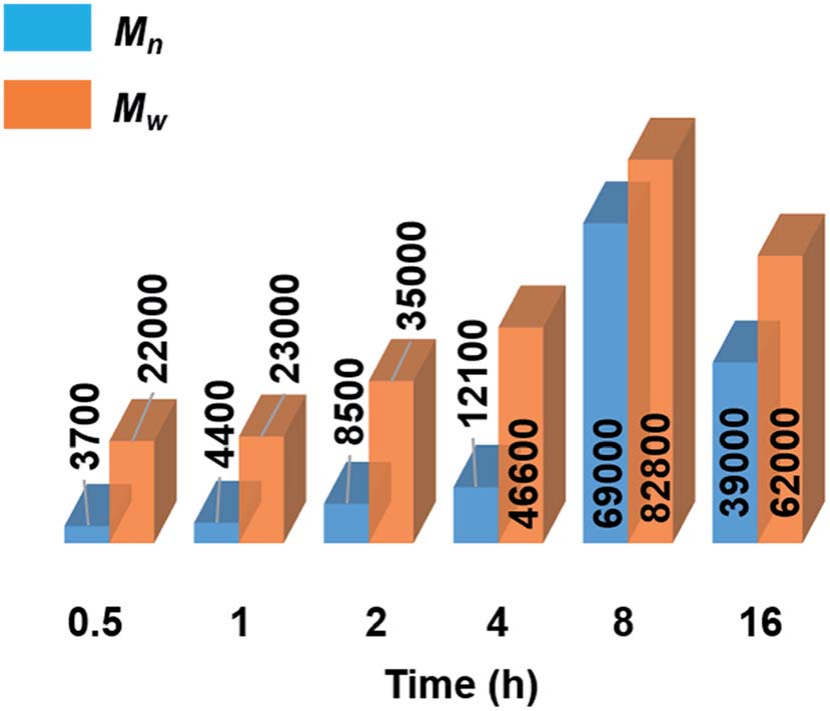
Graphical representation of molecular weights (*M*_n_ and *M*_w_ in g mol^–1^) from GPC analysis of isolated polyaminoborane **5** from the reactions of **4** with precatalyst [**6e** + 2*n*BuLi] (7 mol%, 0.5–16 h, 22 °C) (see Table 2).

Further mechanistic studies were carried out on the dehydropolymerisation of MeNH_2_·BH_3_ (**4**) using 7 mol% of [**6e** + 2*n*BuLi]. We studied the effect of reaction time in more detail by isolating polyaminoborane **5** after 0.5, 1, 2, and 4 h (see Fig. S34–S37†), in addition to the 8 and 16 h time points already recorded. A steady increase in *M*_n_ and a concomitant decrease in PDI of **5** with increasing reaction time up to the 8 h time point was observed (see Fig. 3, S38,† and Table 2). The observation of a decrease in molar mass and increased PDI at prolonged (8–16 h) reaction times was attributed to depolymerisation and dehydrogenation to afford **8** and **9** (Fig. S39†). Similar observations have been reported with [CpFe(CO)_2_]_2_ (ref. 6b) as a precatalyst, whilst this effect was much less significant in the case of [IrH_2_(POCOP)].4*c* We also found that cyclotriborazane **7**, which is likely formed as an intermediate during the depolymerisation of **5**, was rapidly dehydrogenated by [**6e** + 2*n*BuLi] (1 h, toluene, 22 °C) to yield borazine **8** (see Fig. S40†). Interestingly, both the Ti- and Ir-catalysed dehydropolymerisations showed an increase in *M*_n_ with catalyst loading. In the case of the Ir precatalyst, this observation was tentatively interpreted in terms of a chain-growth mechanism that involved an initial, rate-determining dehydrogenation step to form transient MeNH=BH_2_, followed by coordination polymerisation to form **5**.4*c* As for the Ir-catalysed reaction,^28^ attempts to trap the highly reactive MeNH=BH_2_ using cyclohexene,^29^ to form MeNH=BCy_2_, were unsuccessful in the case of the Ti precatalyst (see Fig. S41†). This suggests that if the primary aminoborane is indeed formed as an intermediate, it either remains coordinated or is consumed more rapidly than it undergoes hydroboration with the cyclic olefin.

**Table 2:** Substrate conversion (from ^11^B NMR spectroscopy) and molecular weights (from GPC) for **5** from the reaction of **4** with precatalyst [**6e** + 2*n*BuLi] (7 mol%, 0.5–8 h, 22 °C) in toluene

Time (h)	Conversion of **4** (%)	Molecular weight *M*_n_ (g mol^–1^)	Molecular weight *M*_w_ (g mol^–1^)	PDI
0.5	80 (ref. 31)	3700	22 000	6.2
1	88 (ref. 31)	4400	23 000	5.0
2	90	8500	35 000	4.0
4	92	12 100	46 600	3.8
8	97	54 000	85 000	1.6

Significantly, in the Ti-catalysed polymerisation a steady increase in molar mass was observed from 0.5 h (conversion of **4** = 80%, *M*_n_ = 3700 g mol^–1^, PDI = 6.2) up to 8 h (conversion = 97%, *M*_n_ = 54 000 g mol^–1^, PDI = 1.6) before depolymerisation and dehydrogenation of the polyaminoborane **5** were detected (Table 2) .^30^ This is indicative of a step-growth polycondensation process and contrasts with the behavior found for the dehydropolymerisation of **4** with [IrH_2_(POCOP)] as precatalyst. In the latter case high molar mass **5** was detected even at low conversions of **4**, as befits a chain-growth mechanism.4*c*

The existence of a step-growth polymerisation mechanism for the Ti-catalysed dehydropolymerisation of **4** is consistent with the intermediacy of linear diborazane **2** in the dehydrogenation of **1**. It is also supported by several further experiments. For example, treatment of isolated, low molar mass **5** (*M*_n_ = 2600 g mol^–1^, PDI = 4.3) with a further quantity of 7 mol% of [**6e** + 2*n*BuLi] in toluene for 7.5 h afforded higher molar mass **5** (*M*_n_ = 18 000 g mol^–1^, PDI = 1.8), which demonstrates that monomer is not required to form high molar mass polymer (see Fig. S42 and S43†).^32^ Consistent with the hypothesis that the Ti- and Ir-catalysed polymerisations proceed *via* fundamentally different mechanisms, the molar mass of **5** (*M*_n_ = 3100 g mol^–1^, PDI = 2.7) only increased marginally (*M*_n_ = 6700 g mol^–1^, PDI = 2.5) upon treatment with [IrH_2_(POCOP)], (see Fig. S44 and S45†), whereas under these conditions, the Ir precatalyst converts **4** to **5** with a *M*_n_ of 262 600 g mol^–1^ (PDI = 1.7) (Fig. S47†).

## Conclusions

In summary, we have successfully optimised the precatalyst system for secondary amine–boranes based on Cp^R^_2_TiCl_2_/2*n*BuLi by systematic variation of the cyclopentadienyl ligand steric and electronic properties. Based on these results and with an extension to primary amine–boranes, we report the first example of an early transition metal-mediated synthesis of high molar mass polyaminoboranes *via* dehydropolymerisation of *N*-methyl and *N*-benzyl (and related) substituted amine–boranes. The presented precatalyst system, based on earth abundant titanium, was shown to augment the amine–borane substrate scope exhibited by state-of-the art catalysts, *e.g.* Brookhart's iridium catalyst, skeletal nickel or [Rh(COD)Cl]_2_. Further investigations into the mechanistic pathway for the dehydropolymerisation of MeNH_2_·BH_3_ suggested that it proceeds by a step-growth rather than a chain-growth mechanism.

Previously, the catalytic dehydropolymerisation of intrinsically polar primary amine–borane substrates has required mid to late transition metal centers. It is interesting to note that, in the case of olefins, the analogous developments occurred historically in the reverse order, starting with early metals before the more recent successful development of late transition metal catalysts.

## Conflicts of interest

The authors declare no competing financial interests.

## Supplementary Material

Supplementary information
