# Supplementary material for: Step-growth titanium-catalysed dehydropolymerisation of amine–boranes
Source: Chem Sci. 2018 Mar 6;9(13):3360–6. doi: 10.1039/c7sc05395a (PMC5933219; doi:10.1039/c7sc05395a)
Supplement: Supplementary file 1 [file SC-009-C7SC05395A-s001.pdf]

Electronic Supplementary Material (ESI) for Chemical Science.
This journal is © The Royal Society of Chemistry 2018

Step-Growth Titanium-Catalysed Dehydropolymerisation of Amine-Boranes

Titel Jurca,[a] Theresa Dellermann,[a] Naomi E. Stubbs,[a] Diego A. Resendiz-Lara,[a]
George R. Whittell[a]* and Ian Manners[a]*

Supporting Information

Table of Contents

General Procedures, Reagents, and Equipment: **S2**

Catalytic dehydrocoupling of $Me_2NH·BH_3$ (1) by precatalysts 6a-e: **S2-S7**

Catalytic dehydropolymerisation of $MeNH_2·BH_3$ (4) by precatalysts 6d,e: **S7-S9**

Catalytic dehydropolymerisation of $MeNH_2·BH_3$ (4) by precatalyst 6e (*variable cat. %*): **S9-S14**

Kinetic studies on the catalytic dehydropolymerisation of $BzNH_2·BH_3$ (10a) by precatalyst 6e: **S14-S15**

Catalytic dehydropolymerisation of $RNH_2·BH_3$ (R = CH_2Ph (10a), $(CH_2)_4Ph$ (10b), $CH_2(C_5H_3S)$ (10c)) by precatalyst 6e: **S15-S19**

Catalytic dehydropolymerisation of $BzNH_2·BH_3$ (10a) and *n*$BuNH_2·BH_3$ by precatalyst 6e: **S20-S22**

Catalytic dehydropolymerisation of $MeNH_2·BH_3$ (4) by precatalyst 6e (*variable time*): **S23-S25**

Depolymerisation $[MeNH\text{-}BH_2]_n$ (5) by precatalyst 6e: **S26**

Catalytic dehydrogenation of $[MeNH\text{-}BH_2]_3$ (7) by precatalyst 6e: **S27**

Cyclohexene trapping experiments: **S27-S28**

Catalytic polycondensation of $[MeNH\text{-}BH_2]_n$ (5) by precatalyst 6e: **S28-S30**

Catalytic polycondensation of $[MeNH\text{-}BH_2]_n$ (5) by $[IrH_2(POCOP)]$: **S30-S31**

Catalytic dehydropolymerisation of $MeNH_2·BH_3$ (4) by $[IrH_2(POCOP)]$: **S31-S32**

References: **S32**

General Procedures, Reagents, and Equipment: All manipulations were carried out under an atmosphere of nitrogen gas using standard vacuum line and Schlenk techniques, or under an atmosphere of argon or nitrogen within MBraun gloveboxes. All solvents were dried *via* a Grubbs design solvent purification system.[1] Toluene-d_8 and THF-d_8 (anhydrous) were purchased from Aldrich and stored over activated molecular sieves. $Me_2NH·BH_3$ (97%) (**1**), was purchased from Aldrich and further purified by vacuum sublimation twice at 20 °C. $MeNH_2·BH_3$ (**4**), $BzNH_2·BH_3$ (**10a**), $Ph(CH_2)_4NH_2·BH_3$ (**10b**), $(C_5H_3S)CH_2NH_2·BH_3$ (**10c**), nBuNH$_2$·BH$_3$ (**10d**) and samples of $[MeNH\text{-}BH_2]_n$ (**5**), and $[MeNH\text{-}BH_2]_3$ (**7**), were synthesised according to literature procedure.[2-5] Titanium precatalysts **6a**,**c-e** were purchased from Aldrich and used as received. Precatalysts **6b** was synthesised according to literature procedures.[6,7] NMR spectra were recorded using a Jeol ECP(Eclipse) 300 or a Bruker Avance III HD 500 Cryo spectrometer. Chemical shifts are reported calibrated against residual ^1H and ^{13}C resonances of the respective deuterated solvent relative to tetramethylsilane (^{13}C, ^1H) or to internal standard B(O*i*Pr)$_3$ (^{11}B). Integration of ^{11}B NMR spectra was performed using MestReNova Version 7.1.1-9649 with an estimated accuracy of ± 5%. Gel permeation chromatography (GPC) was performed on a Viscotek RImax chromatograph, equipped with an automatic sampler, a pump, an injector and inline degasser. The columns were contained within an oven (35 °C) and consisted of styrene/divinyl benzene gels with pore sizes ranging from 500 Å to 100,000 Å. THF containing 1 % w/w [nBu$_4$N]Br and 1% v/v toluene was used as the eluent at a flow rate of 1.0 mL min^{-1}. All samples analysed by GPC were dissolved in the eluent (2 mg mL^{-1} in THF), stirred for 1 h at room temperature and passed through a membrane filter (200 nm pores) before analysis. The calibration was conducted using a series of monodisperse polystyrene standards obtained from Aldrich. The elemental analysis was carried out on a CE Instruments (now Thermo) elemental analyser model EA1110. Mass spectra was obtained on a Water Synapt G2S instrument equipped with a nanospray ionisation module using a spray voltage of 1.5 kV (Advion TriVersa Nanomate). Samples were prepared as CH_2Cl_2/THF solutions (conc. 1 mg/ml).

Catalytic dehydrocoupling of $Me_2NH·BH_3$ (1) by precatalysts 6a-e. To a solution of substrate **1** (60 mg, 1.0 mmol), in 0.7 mL of anhydrous toluene-d_8 (anhydrous THF-d_8) was added a solution of precatalyst [**6a,c-e** + 2nBuLi] (0.02 mmol) in 0.3 mL toluene-d_8 (anhydrous THF-d_8). For precatalyst **6b**, the procedure was identical, except no nBuLi was required. A 0.5 mL aliquot of the solution was then charged into a quartz J Young NMR tube with approximately 1.7 mL of headspace and a sealed capillary of B(O*i*Pr)$_3$ as standard, then sealed and allowed to react at 22 °C. Solutions gradually changed from a dark green to a dark indigo colour, and formation of H_2 was immediately noticeable for all precatalysts; readily observed by formation of bubbles and substantial pressure release upon opening the NMR tube post reaction. Reactions were monitored by $^{11}B\{^1H\}$ NMR (96 MHz) spectroscopy for the formation of products **2** ($\delta^{11}B$ -13.8 ppm for terminal BH_3 moiety and 1.6 ppm for internal BH_2 moiety), and **3** ($\delta^{11}B$ 4.9 ppm). Also present in minor amounts were diaminoborane $(Me_2N)_2BH$ ($\delta^{11}B$ 28.4 ppm) and

aminoborane $Me_2N{=}BH_2$ ($\delta^{11}B$ 37.4 ppm). Spectra were acquired at 5 min intervals for the first 60 min, followed by acquisitions at 30 min intervals for a total of 690 min. (11.5 h). See figures S1-S6.

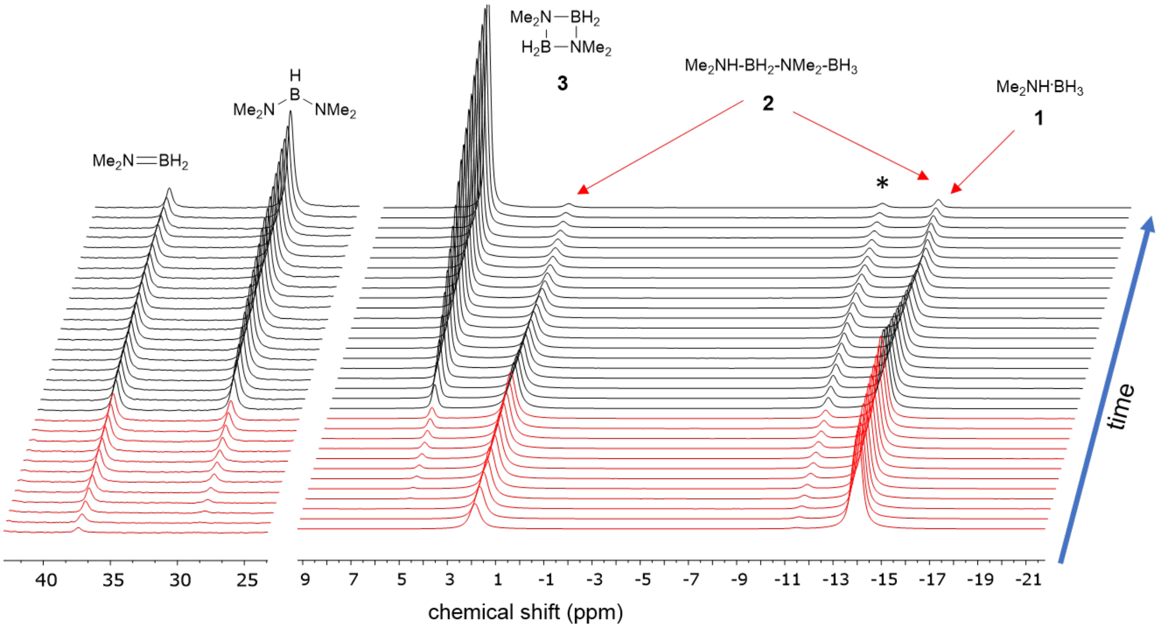

Figure S1: $^{11}B\{^1H\}$ (96 MHz, toluene-d_8) NMR spectra showing (*right*) conversion of **1** to **2** and **3** by precatalyst [**6a** + 2*n*BuLi] (2 mol%, 22 °C), and (*left*) concomitant appearance of minor products $Me_2N{=}BH_2$ and $(Me_2N)_2BH$. Note, the first 12 spectra (***red***) were acquired at 5 min intervals, the remainder (***black***) were acquired at 30 min intervals. * = *very small amount of unidentified species at δ -11.6 ppm, as previously reported for reactions utilizing **6a** (+ 2nBuLi) as precatalyst.*[8]

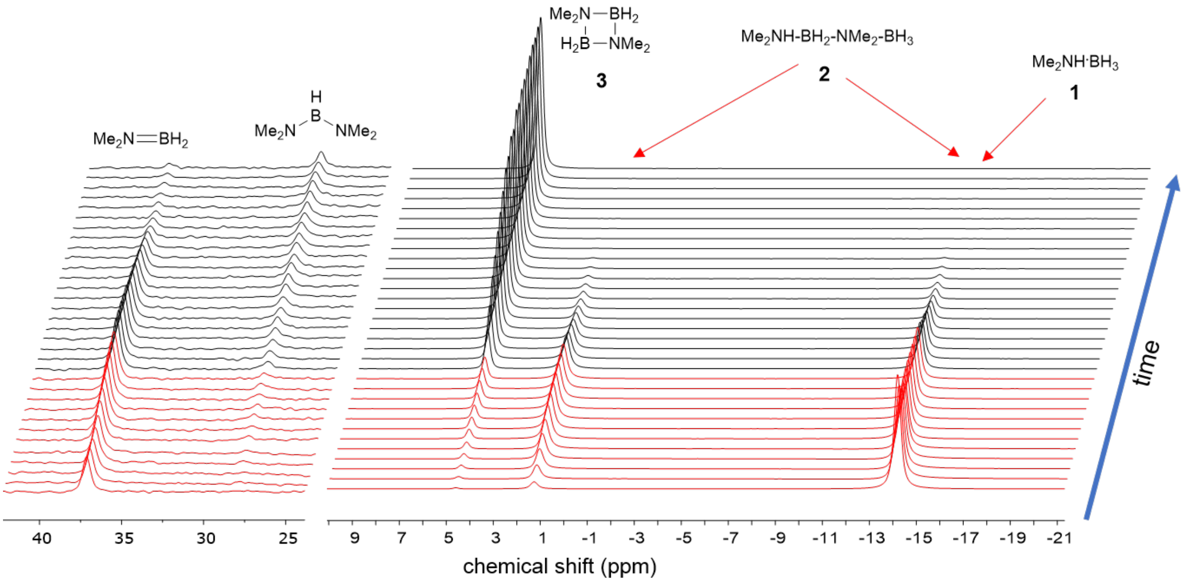

Figure S2: $^{11}B\{^1H\}$ (96 MHz, toluene-d_8) NMR spectra showing (*right*) conversion of **1** to **2** and **3** by precatalyst **6b** (2 mol%, 22 °C), and (*left*) concomitant appearance of minor products $Me_2N{=}BH_2$ and $(Me_2N)_2BH$. Note, the first 12 spectra (***red***) were acquired at 5 min intervals, the remainder (***black***) were acquired at 30 min intervals.

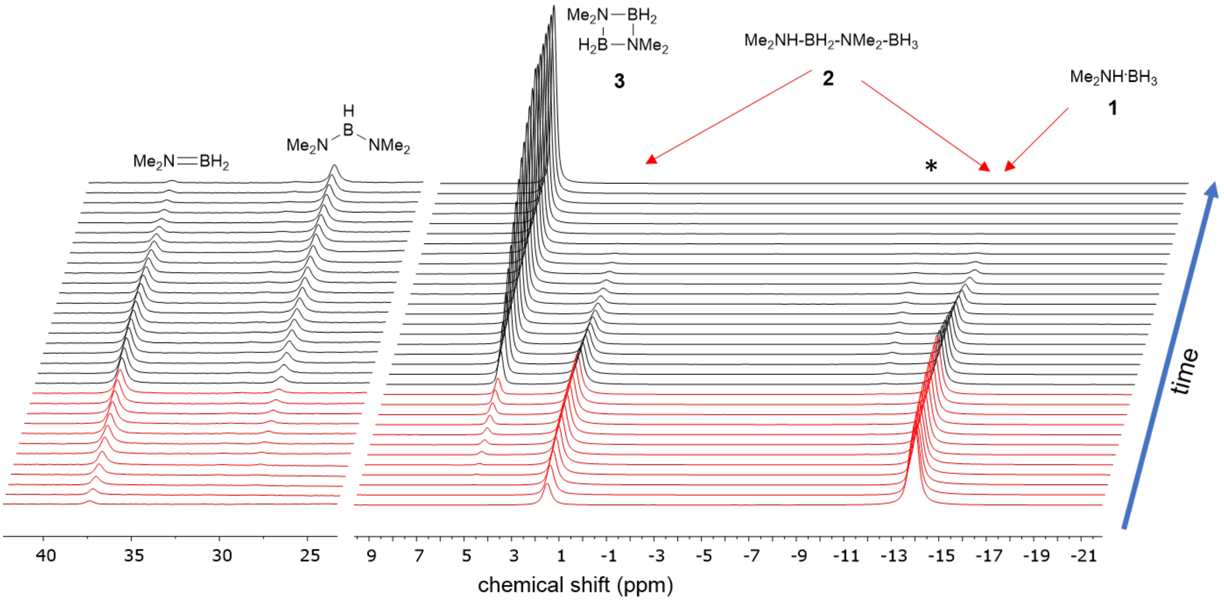

Figure S3: $^{11}B\{^1H\}$ (96 MHz, toluene-d_8) NMR spectra showing (*right*) conversion of **1** to **2** and **3** by precatalyst [**6c** + 2*n*BuLi] (2 mol%, 22 °C), and (*left*) concomitant appearance of minor products $Me_2N{=}BH_2$ and $(Me_2N)_2BH$. Note, the first 12 spectra (***red***) were acquired at 5 min intervals, the remainder (***black***) were acquired at 30 min intervals. * = *very small amount of unidentified species at δ -11.6 ppm, as previously reported for reactions utilizing* ***6a*** *(+ 2nBuLi) as precatalyst.*[8]

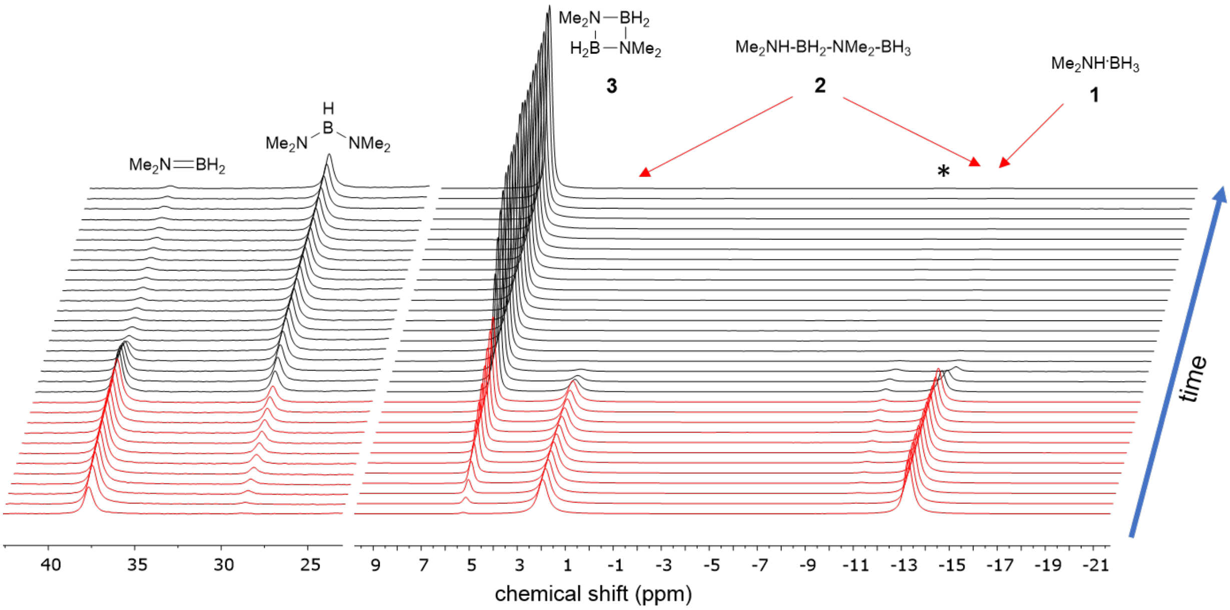

Figure S4: $^{11}B\{^1H\}$ (96 MHz, toluene-d_8) NMR spectra showing (*right*) conversion of **1** to **2** and **3** by precatalyst [**6d** + 2*n*BuLi] (2 mol%, 22 °C), and (*left*) concomitant appearance of minor products $Me_2N{=}BH_2$ and $(Me_2N)_2BH$. Note, the first 12 spectra (***red***) were acquired at 5 min intervals, the remainder (***black***) were acquired at 30 min intervals. * = *very small amount of unidentified species at δ -11.6 ppm, as previously reported for reactions utilizing* ***6a*** *(+ 2nBuLi) as precatalyst.*[8]

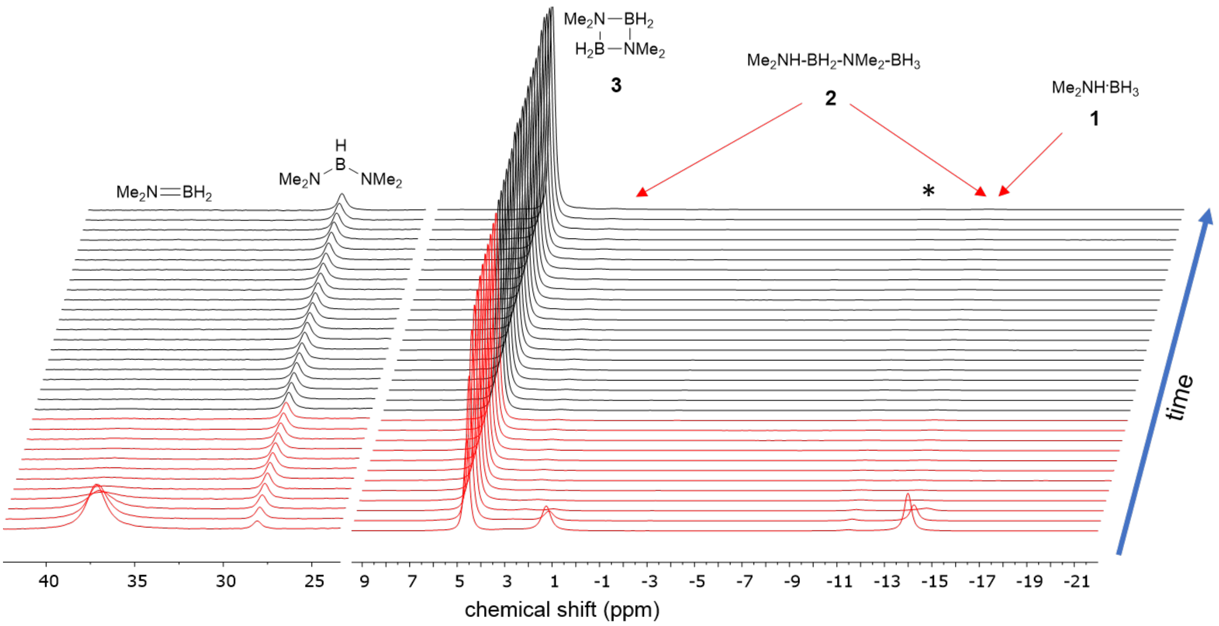

Figure S5: $^{11}B\{^{1}H\}$ (96 MHz, toluene-d_8) NMR spectra showing (*right*) conversion of **1** to **2** and **3** by precatalyst [**6e** + 2*n*BuLi] (2 mol%, 22 °C), and (*left*) concomitant appearance of minor products $Me_2N{=}BH_2$ and $(Me_2N)_2BH$. Note, the first 12 spectra (***red***) were acquired at 5 min intervals, the remainder (***black***) were acquired at 30 min intervals. * = *very small amount of unidentified species at δ -11.6 ppm, as previously reported for reactions utilizing* ***6a*** *(+ 2nBuLi) as precatalyst.*[8]

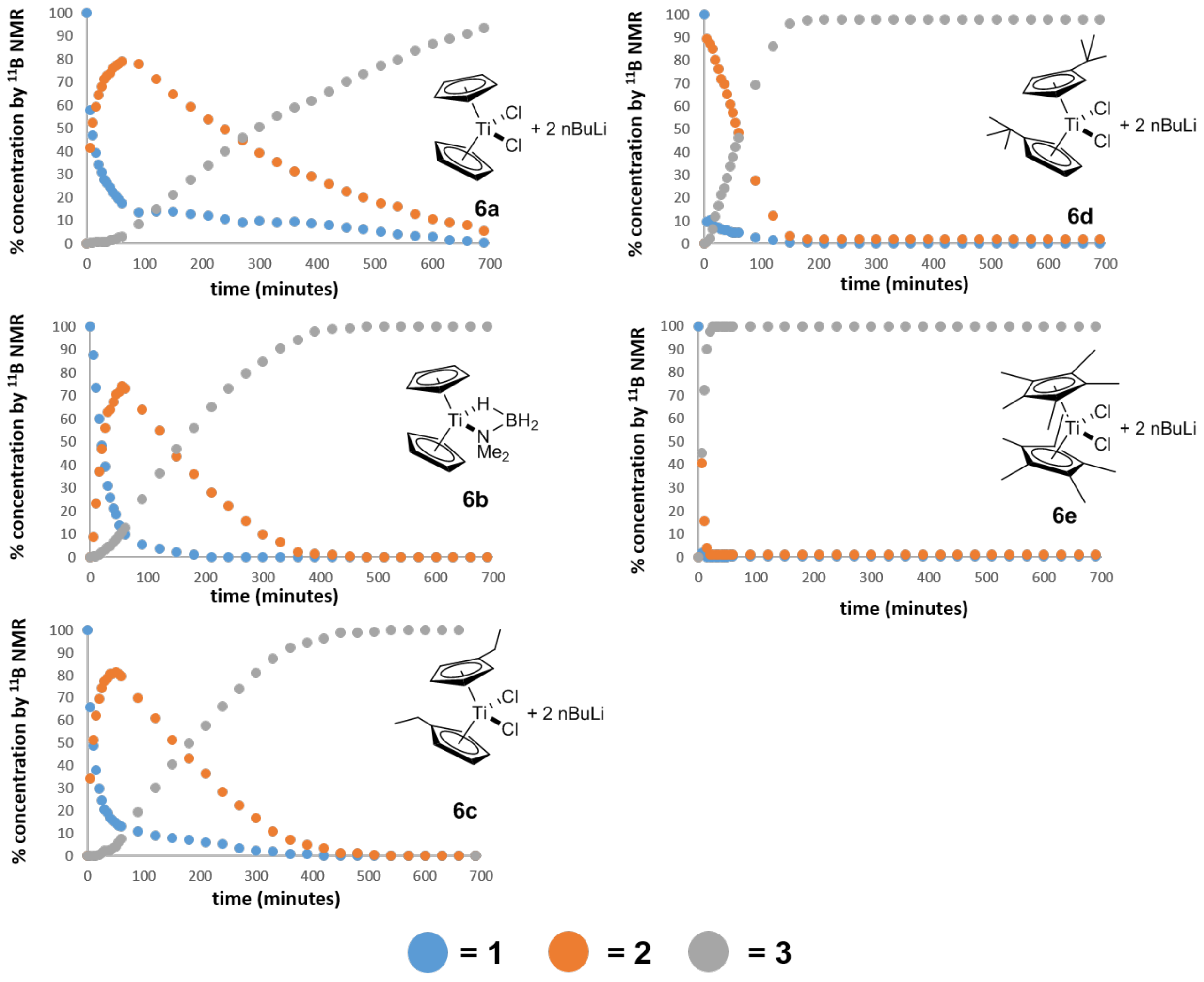

Figure S6: Reaction profiles for the formation of **3** from the catalytic dehydrocoupling of **1**, *via* **2** with precatalysts [**6a,c-e** + 2*n*BuLi], and **6b** as monitored by $^{11}B\{^{1}H\}$ (96 MHz, toluene-d_8) NMR spectroscopy.

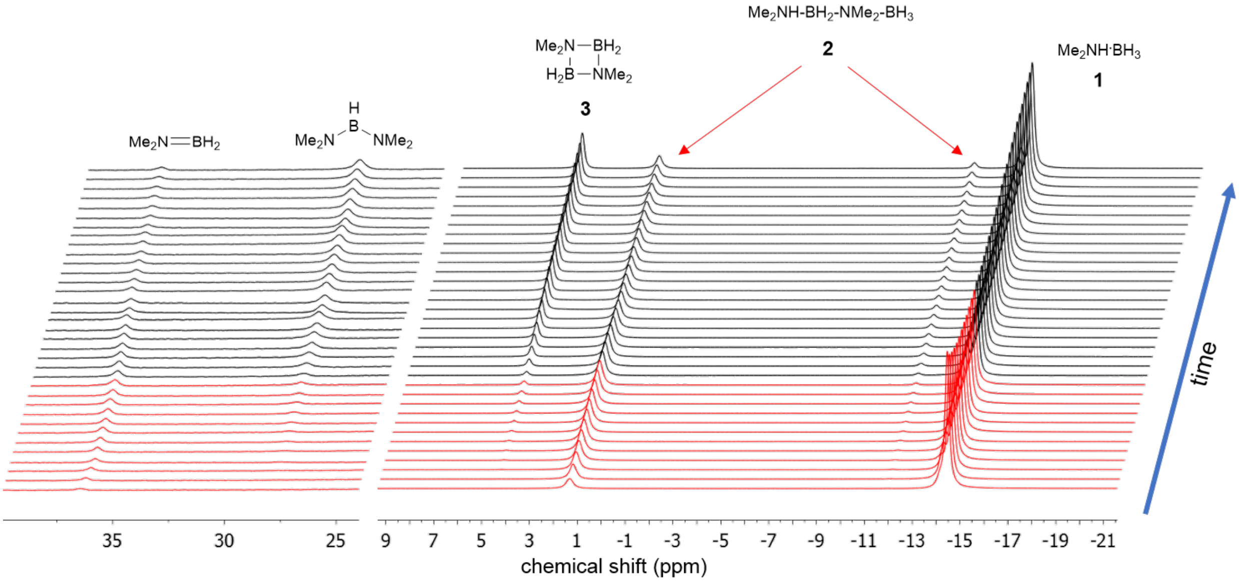

Figure S7: $^{11}B\{^1H\}$ (96 MHz, THF-d_8) NMR spectra showing (*right*) conversion of **1** to **2** and **3** by precatalyst [**6e** + 2*n*BuLi] (2 mol%, 22 °C) in THF, and (*left*) concomitant appearance of minor products Me_2N=BH_2 and $(Me_2N)_2BH$. Note, the first 12 spectra (***red***) were acquired at 5 min intervals, the remainder (***black***) were acquired at 30 min intervals.

Table S1: Turnover Frequency (TOF) calculations, measured below 50% conversion. TOF = [(moles of **3**)/(moles of catalyst)]/(reaction time); *moles of catalyst* = 0.00002.

precatalyst	*% conc. **3** by ^{11}B NMR*	*moles of **3***	*time (h)*	*TOF (h^{-1})*
***6a** + 2nBuLi*	*48*	*0.00024*	*4.50*	*2.7*
6b	*47*	*0.000235*	*2.50*	*4.7*
***6c** + 2nBuLi*	*48*	*0.00024*	*2.50*	*4.8*
***6d** + 2nBuLi*	*46*	*0.00023*	*1.00*	*11.5*
***6e** + 2nBuLi*	*45*	*0.000225*	*0.08*	*141*

Catalytic dehydropolymerisation of $MeNH_2{\cdot}BH_3$ (4) by precatalysts 6d,e (*NMR experiments*). To a solution of substrate **4** (45 mg, 1.0 mmol), in 0.7 mL of anhydrous toluene-d_8 was added a solution of precatalyst [**6d,e** + 2*n*BuLi] (0.02 mmol) in 0.3 mL toluene-d_8. A 0.5 mL aliquot of the solution was then charged into a quartz J Young NMR tube with approximately 1.7 mL of headspace and a sealed capillary of $B(OiPr)_3$ as standard, then sealed and allowed to react at 22 °C. Solutions gradually changed from a dark green to a dark indigo/brown colour, and formation of gas was immediately noticeable for both precatalysts. Reactions were monitored by $^{11}B\{^1H\}$ NMR (96 MHz) spectroscopy for the formation of product **5** ($\delta^{11}B$ -6.1 ppm) with the small peak at $\delta^{11}B$ -18 ppm assigned to the polymer end-group $[MeNH$-$BH_2]_n$-NMeH-*B*H_3 (see discussion in main text). The presence of small amounts of unreacted **4** in the reaction mixture, however, cannot be excluded). Also present were varying amounts of byproducts $[MeNH$-$BH_2]_3$, **7** ($\delta^{11}B$ -5.8 ppm), $[MeN$-$BH]_3$, **8** ($\delta^{11}B$ 32.5 ppm), $(MeNH)_2BH$, and **9** ($\delta^{11}B$ 27.7 ppm).

Spectra were acquired at 5 min intervals for the first 60 min, followed by acquisitions at 30 min intervals for a total of 720 min (12 h), and for precatalyst **6e**, additionally at 23 h. See Figures S9 and S10.

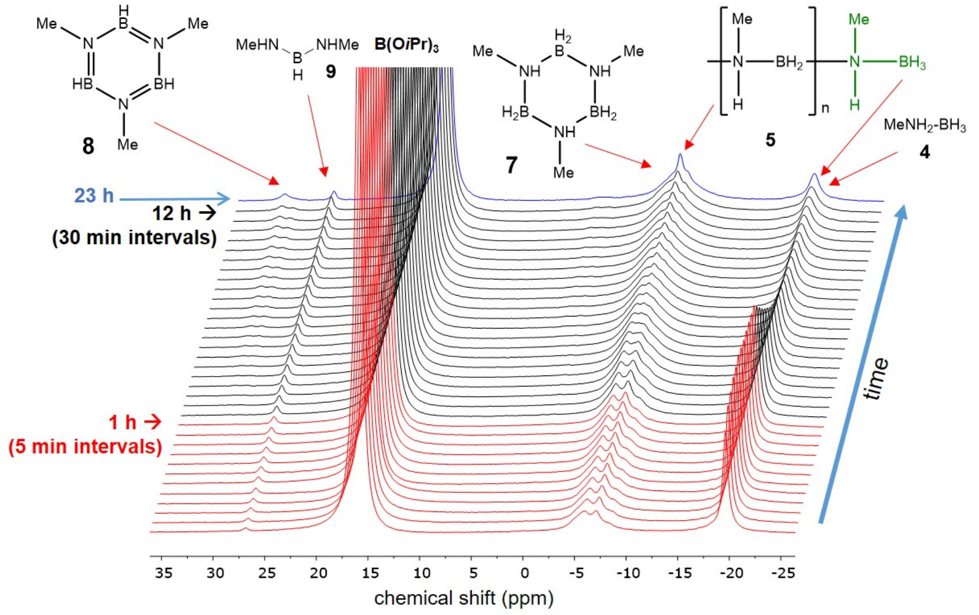

Figure S8: $^{11}B\{^1H\}$ (96 MHz, toluene-d_8) NMR spectra showing conversion of **4** to **5** with byproducts **7**, **8**, **9** and trace MeNH=BH_2 utilizing precatalyst [**6d** + 2*n*BuLi] (2 mol%, 22 °C). *Note: 7 possess a sharp peak at $\delta^{11}B$ -5.8 ppm which is superimposed on the broad peak for **5**. The broad signal at $\delta^{11}B$ -18 ppm is assigned to the polymer end-group of **5**, [MeNH-BH_2]$_n$-NMeHBH_3, by reference to later purified material (see Figures S11-18). The presence of some unreacted **4**, however, cannot be excluded.*

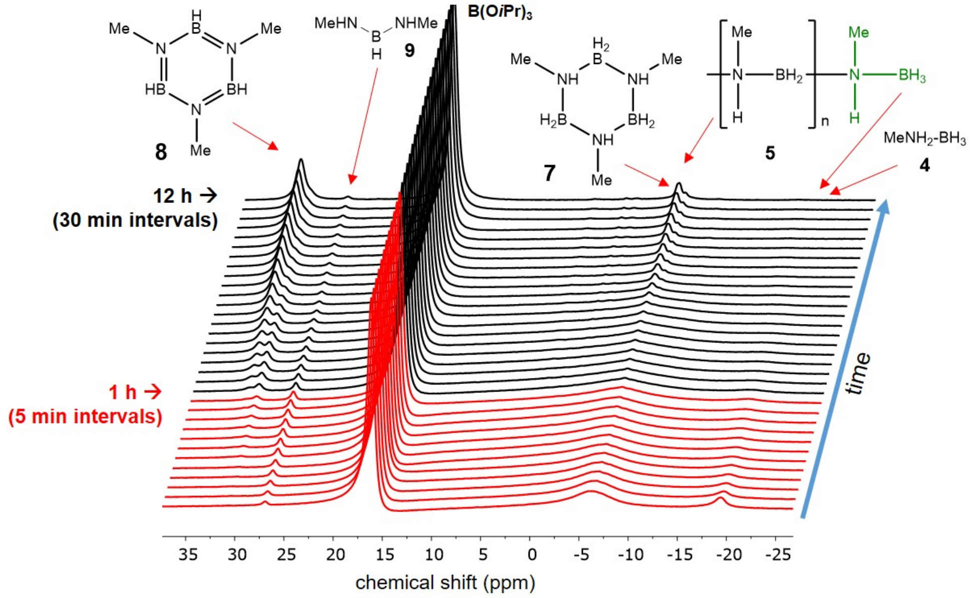

Figure S9: $^{11}B\{^1H\}$ (96 MHz, toluene-d_8) NMR spectra showing conversion of **4** to **5** with byproducts **7**, **8**, **9** and trace MeNH=BH_2 utilizing precatalyst [**6e** + 2*n*BuLi] (2 mol%, 22 °C). *Note: 7 possess a sharp peak at $\delta^{11}B$ -5.8 ppm which is superimposed on the broad peak for **5**. The broad signal at $\delta^{11}B$ -18 ppm is assigned to the polymer end-group of **5**, [MeNH-BH_2]$_n$-NMeH-BH_3, by reference to later purified material (see Figures S11-18). The presence of some unreacted **4**, however, cannot be excluded.*

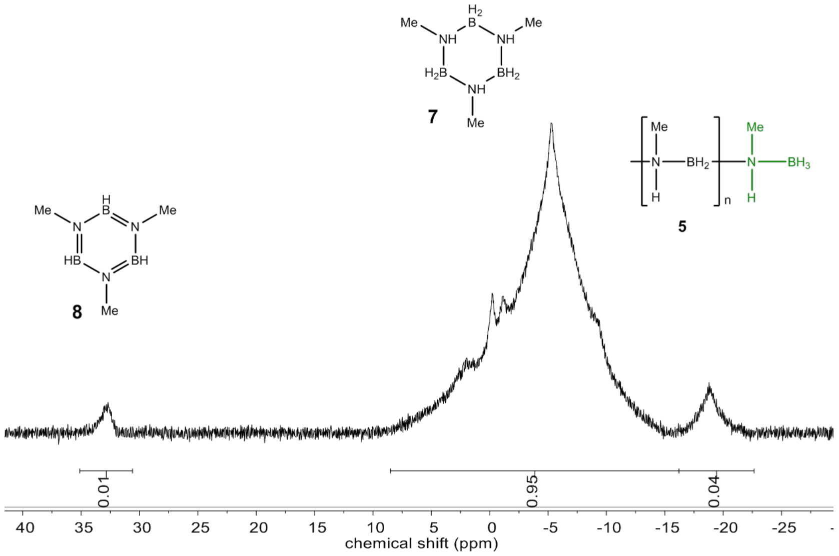

Figure S10 $^{11}B\{^{1}H\}$ (96 MHz, THF-d_8) NMR spectra showing conversion of **4** to **5** with byproducts **7** and **8** after 8 h utilizing precatalyst [**6e** + 2*n*BuLi] (2 mol%, 22 °C). *Note: 7 possess a sharp peak at* $\delta^{11}B$ *-5.8 ppm which is superimposed on the broad peak for* ***5****. The broad signal at* $\delta^{11}B$ *-18 ppm is assigned to the polymer end-group of* ***5****,* $[MeNH\text{-}BH_2]_n\text{-}NMeH\text{-}BH_3$*, (see main text and Figures S11-18). The presence of small amounts of unreacted* ***4****, however, cannot be excluded.*

Catalytic dehydropolymerisation of $MeNH_2{\cdot}BH_3$ (4) by precatalyst 6e (*variable catalyst loading*). To a solution of substrate **4** (135 mg, 3.0 mmol), in 1-1.94 mL of anhydrous toluene was added a solution of precatalyst [**6e** + 2*n*BuLi] (0.012-0.21 mmol) in 0.06-1 mL toluene (Total volume = 2 mL); corresponding to catalyst loadings of 0.4, 2, 4 and 7% respectively. The solution was allowed to stir at 22 °C for 8 h or 16 h. The colour gradually changed from a dark green to a dark indigo/brown, and formation of gas was immediately noticeable. Upon completion, the reaction solution was transferred dropwise to vigorously stirred *n*-hexanes at -40 °C, and a white solid precipitated. The solid was filtered off and dried under vacuum (yield 53-72%). As expected from the *in situ* studies, $^{11}B\{^{1}H\}$ NMR (96 MHz) spectroscopy revealed the formation of product **5** ($\delta^{11}B$ -6.1 ppm), with the observed peak at *ca.* $\delta^{11}B$ -18 ppm assigned to the polymer end-group $[MeNH\text{-}BH_2]_n\text{-}NMeH\text{-}BH_3$ (see discussion in main text). The presence of small amounts of unreacted **4** in the reaction mixture, however, cannot be excluded. In the instance of precatalyst [**6e** + 2*n*BuLi] (7 mol%, 16 h, 22 °C), $[MeNH\text{-}BH_2]_3$, **7** ($\delta^{11}B$ -5.8 ppm) was also present (see Figure S18). The very small shoulder at $\delta^{11}B$ +2 ppm (Figures S10-13) may be tentatively assigned to $B(NMeH)_4$ moieties arising from chain branching based on previous work.[9] Resulting molecular weights were determined by GPC analysis (Figure S19) and are summarised in Table S2.

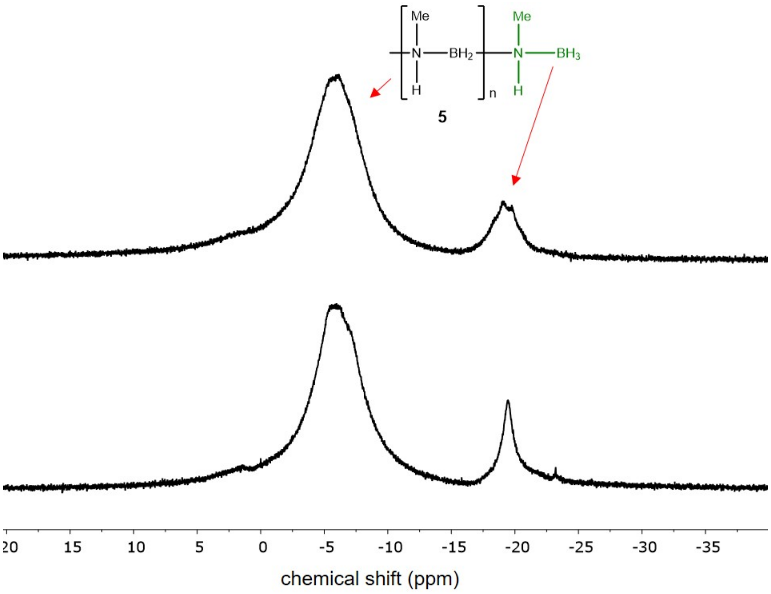

Figure S11: (*bottom*) $^{11}B\{^{1}H\}$ (96 MHz, toluene), (*top*) ^{11}B (96 MHz, toluene) NMR spectra of the isolated product (**5**) from the reaction of **4** with precatalyst [**6e** + 2*n*BuLi] (0.4 mol%, 8 h, 22 °C).

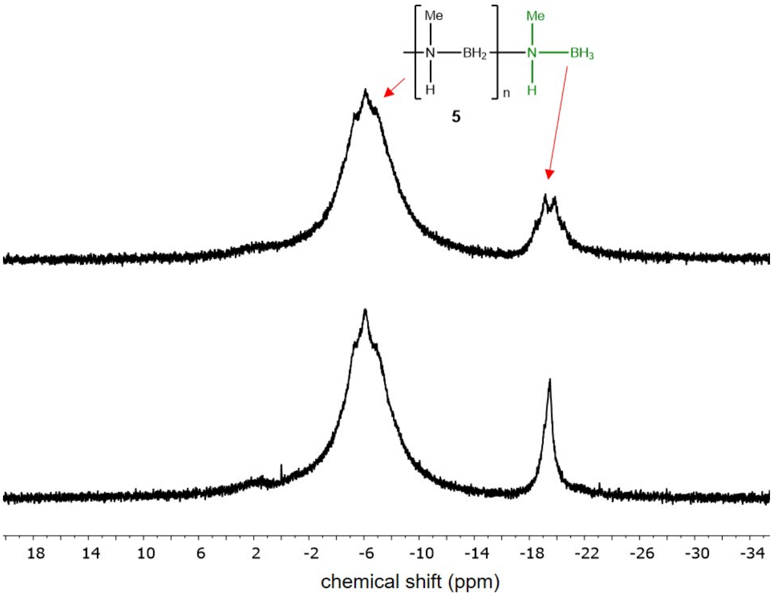

Figure S12: (*bottom*) $^{11}B\{^{1}H\}$ (96 MHz, toluene), (*top*) ^{11}B (96 MHz, toluene) NMR spectra of the isolated product (**5**) from the reaction of **4** with precatalyst [**6e** + 2*n*BuLi] (0.4 mol%, 16 h, 22 °C).

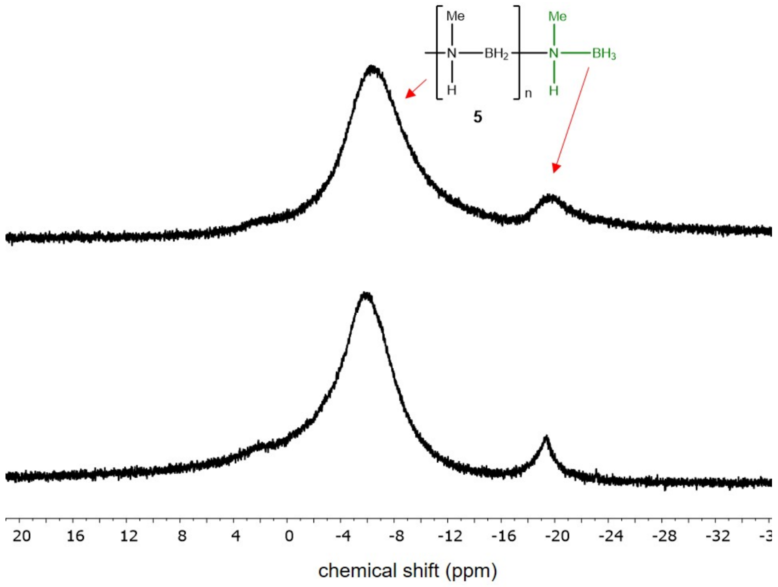

Figure S13: (*bottom*) $^{11}B\{^1H\}$ (96 MHz, toluene), (*top*) ^{11}B (96 MHz, toluene) NMR spectra of the isolated product (**5**) from the reaction of **4** with precatalyst [**6e** + 2*n*BuLi] (2 mol%, 8 h, 22 °C).

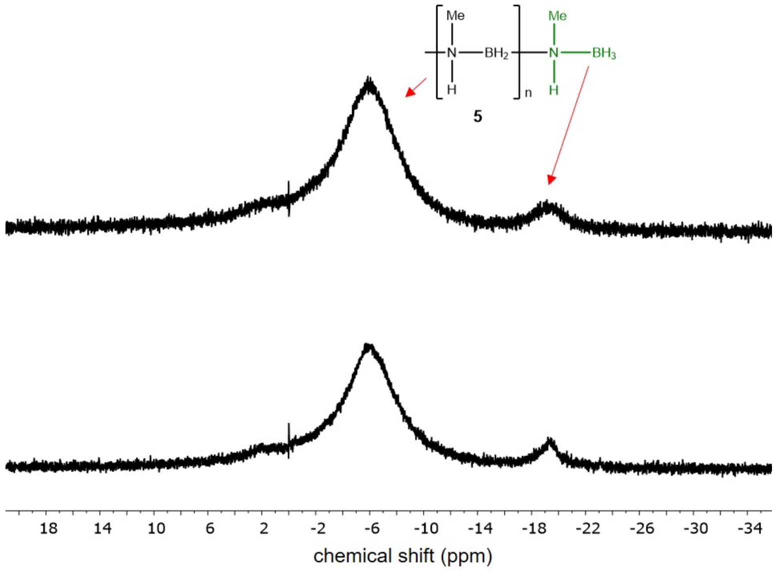

Figure S14: (*bottom*) $^{11}B\{^1H\}$ (96 MHz, toluene), (*top*) ^{11}B (96 MHz, toluene) NMR spectra of the isolated product (**5**) from the reaction of **4** with precatalyst [**6e** + 2*n*BuLi] (2 mol%, 16 h, 22 °C).

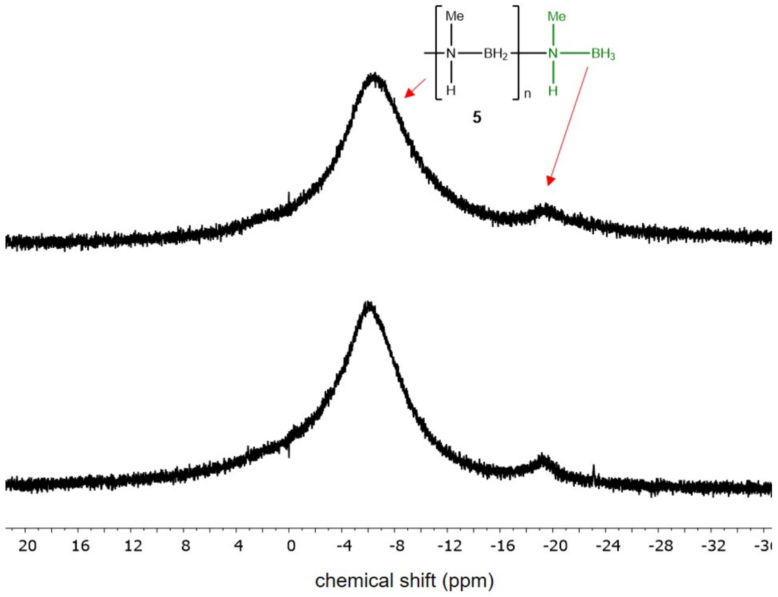

Figure S15: (*bottom*) $^{11}B\{^{1}H\}$ (96 MHz, toluene), (*top*) ^{11}B (96 MHz, toluene) NMR spectra of the isolated product (**5**) from the reaction of **4** with precatalyst [**6e** + 2*n*BuLi] (4 mol%, 8 h, 22 °C).

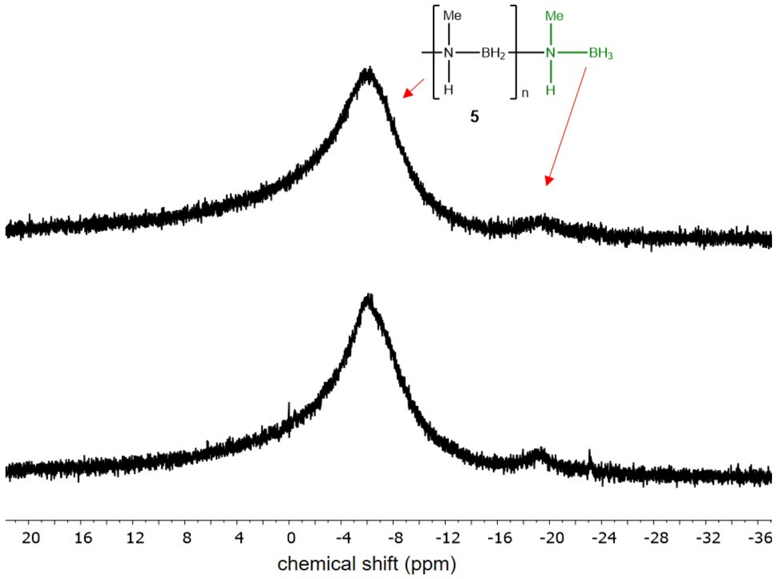

Figure S16: (*bottom*) $^{11}B\{^{1}H\}$ (96 MHz, toluene), (*top*) ^{11}B (96 MHz, toluene) NMR spectra of the isolated product (**5**) from the reaction of **4** with precatalyst [**6e** + 2*n*BuLi] (4 mol%, 16 h, 22 °C).

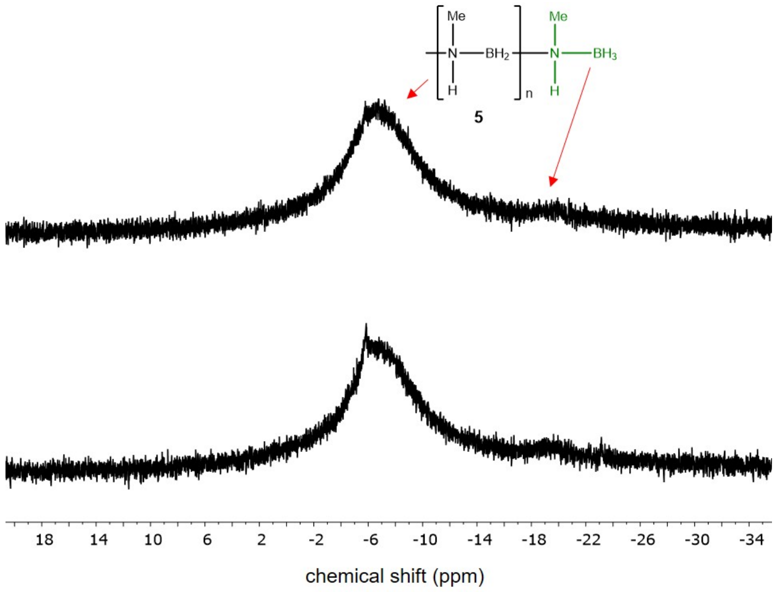

Figure S17: (*bottom*) $^{11}B\{^{1}H\}$ (96 MHz, toluene), (*top*) ^{11}B (96 MHz, toluene) NMR spectra of the isolated product (**5**) from the reaction of **4** with precatalyst [**6e** + 2*n*BuLi] (7 mol%, 8 h, 22 °C).

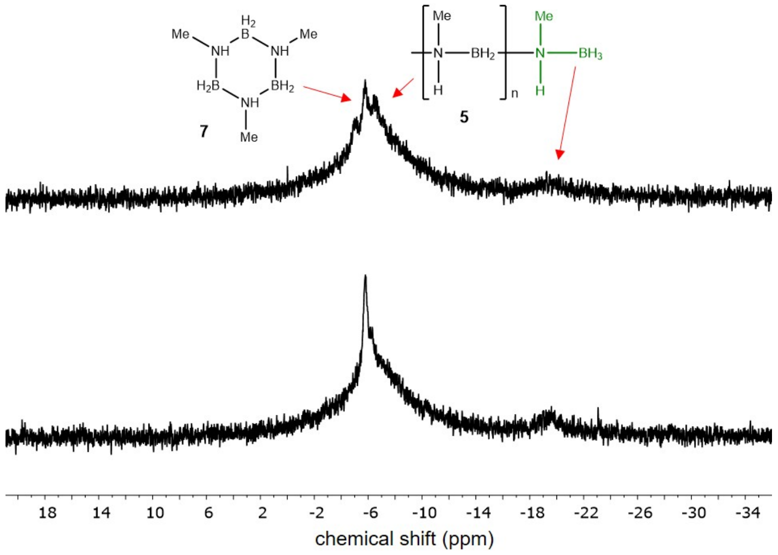

Figure S18: (*bottom*) $^{11}B\{^{1}H\}$ (96 MHz, toluene), (*top*) ^{11}B (96 MHz, toluene) NMR spectra of the isolated product (**5**) from the reaction of **4** with precatalyst [**6e** + 2*n*BuLi] (7 mol%, 16 h, 22 °C). Formation of byproduct **7** is also observed.

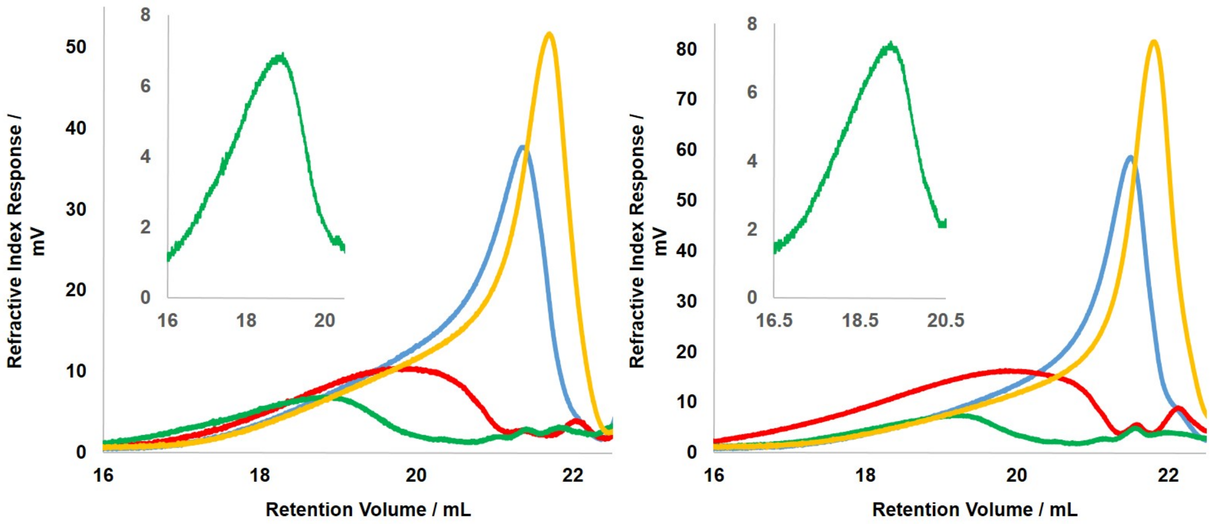

Figure S19: GPC traces for isolated **5** from the reactions of **4** with precatalyst [**6e** + 2*n*BuLi] (0.4, 2, 4 or 7 mol%, 22 °C) for (*left*) 8 h, and (*right*) 16 h. *inse*t = expanded chromatograms for 7 mol % cat. for both 8 h (*left*) and 16 h (*right*) trials. See page S2 for more information.

Table S2: Molecular weights from GPC analysis of **5** from the reactions of **4** with precatalyst [**6e** + 2*n*BuLi] (0.4-7 mol%, 8 h and 16 h, 22 °C).

*[**6e** + 2nBuLi] (mol %)*	*Time (h)*	*Molecular Weight (M_n) (g mol^{-1})*	*PDI*
0.4	*8*	*1 400*	*8.2*
	16	*1 000*	*8.1*
2	*8*	*2 800*	*5.5*
	16	*1 900*	*6.6*
4	*8*	*19 000*	*2.2*
	16	*15 000*	*3.6*
7	*8*	*54 000*	*1.6*
	16	*39 000*	*1.6*

Kinetic studies on the catalytic dehydropolymerisation of $BzNH_2{\cdot}BH_3$ (10a) by precatalyst 6e (*NMR experiment*). To a solution of substrate **10a** (102 mg, 1.0 mmol), in 0.7 mL of anhydrous toluene was added a solution of precatalyst [**6e** + 2*n*BuLi] (0.07 mmol) in 0.3 mL toluene. A 0.5 mL aliquot of the solution was then charged into a quartz J Young NMR tube with approximately 1.7 mL of headspace and a sealed capillary of B(O*i*Pr)$_3$ as standard, then sealed and allowed to react at 22 °C. A formation of gas was immediately noticeable. Reactions were monitored by $^{11}B\{^1H\}$ NMR (96 MHz) spectroscopy for the formation of product **11a** ($\delta^{11}B$ -5.4 ppm with $\delta^{11}B$ -18 ppm assigned to the polymer end-group [BzNH-BH$_2$]$_n$-NBzH-BH$_3$ by reference to spectra of purified material. The presence of unreacted **10** in the

reaction mixture, however, cannot be excluded). During the course of the reaction also varying amounts of byproducts were present $[BzNH\text{-}BH_2]_3$, **12** (δ^{11}B -3.6 ppm), $[BzN\text{-}BH]_3$, **13** (δ^{11}B 32 ppm), $(BzNH)_2BH$, and **14** (δ^{11}B 28 ppm). Spectra were acquired at 5 min intervals for the first 60 min, followed by acquisitions at 30 min intervals for a total of 720 min (12 h). See Figure S20.

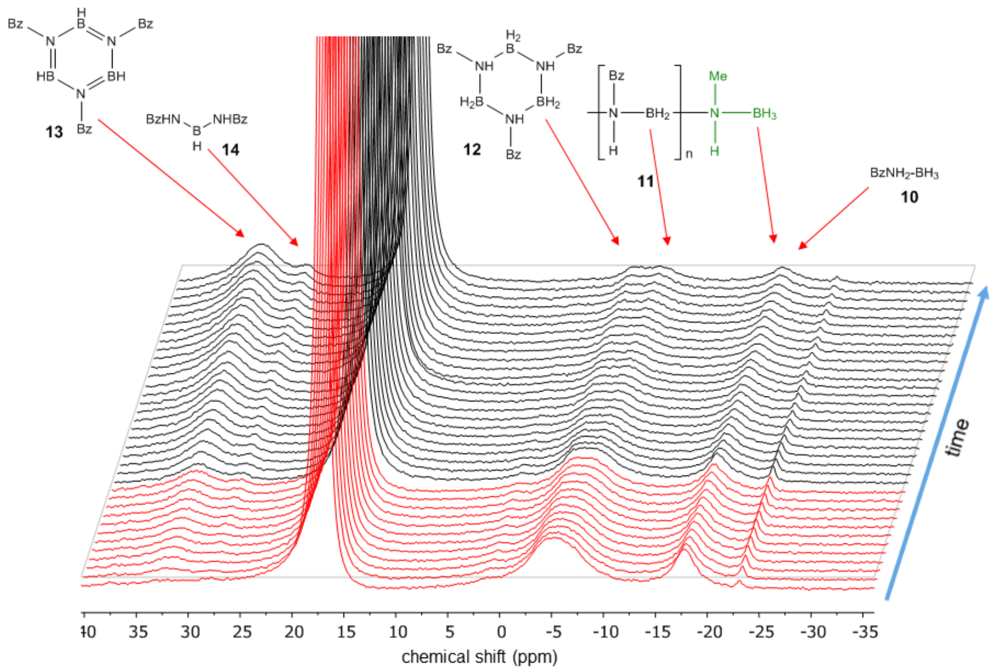

Figure S20: ^{11}B{^{1}H} (96 MHz, toluene-d_8) NMR spectra showing conversion of **10** to **11** with byproducts **12**, **13**, **14** utilizing precatalyst [**6e** + 2*n*BuLi] (7 mol%, 22 °C). *Note: The broad signal at δ^{11}B -19 ppm is assigned to the polymer end-group of **11**, $[BzNH\text{-}BH_2]_n\text{-}NBzH\text{-}BH_3$, by reference to previous described polymer **5**. The presence of some unreacted **10a**, however, cannot be excluded.*

Catalytic dehydropolymerisation of $RNH_2{\cdot}BH_3$ (R = CH_2Ph (10a), $(CH_2)_4Ph$ (10b), $CH_2(C_5H_3S)$ (10c)) by precatalyst 6e (*optimised condition, 7 mol%, 8 h*) yielding homopolymers 11a–11c. To a solution of substrate (**10a**: 102 mg, **10b**: 163 mg, **10c**: 127 mg; 1.0 mmol), in 1 mL of anhydrous toluene was added a solution of precatalyst [**6e** + 2*n*BuLi] (0.07 mmol) in 1 mL toluene. Hydrogen formation could be immediately observed. The solution was allowed to stir at 22 °C for 8 h while the colour gradually changed from a dark green to a dark indigo/brown. Upon completion the reaction solution was transferred dropwise to vigorously stirred *n*-hexanes at room temperature and a white solid precipitated. The solid was filtered off and dried under vacuum (yield **11a**: 31%, **11b**: 61%, **11c**: 44%). Analysis for **11a-c** (Figure S21-S26): **11a**: ^{11}B{^{1}H} NMR (96 MHz, THF-d_8, 22 °C) δ = -9 (br) ppm. Elemental analysis calculated for $C_7H_{10}BN$: C 70.67 %, H 8.47 %, N 11.77 %; found: C 68.37 %, H 8.69 %, N 11.39 %: GPC: M_n = 101 700 g mol^{-1}, PDI = 1.15. **11b**: ^{1}H NMR (500 MHz, THF-d_8, 22 °C) δ = 7.25–6.98 (m, 5 *H* CH_{aryl}) 3.00–2.26 (m, 5 *H*, CH_2, N*H*, B*H*), 2.19–1.20 (m, 6.5 *H*, CH_2, N*H*, B*H*, THF) ppm; ^{13}C NMR (125.8 MHz, THF-d_8, 22 °C) δ = 143.3(C_q), 129.24 (*C*H), 129.17 (*C*H), 126.6 (*C*H), 51.83

(CH_2), 36.78 (CH_2), 30.5 (CH_2), 28.9 (CH_2) ppm; $^{11}B\{^1H\}$ NMR (96 MHz, THF-d_8, 22 °C) δ = -10 (br) ppm; GPC: M_n = 349 100 g mol^{-1}, PDI = 1.30. **11c**: ^1H NMR (500 MHz, CD_2Cl_2, 22 °C) δ = 7.84–5.78 (m, 3 *H* CH_{aryl}), 4.14–3.35 (br, 2 *H*, CH_2), 3.35–2.69 (br, 1 *H*, N*H*), 2.32–1.34 (br, 2 *H*, B*H*) ppm; ^{13}C NMR (125.8 MHz, CD_2Cl_2, 22 °C) δ = 150.7 (C_q), 142.2 (*C*H), 138.8 (C_q), 128.7 (*C*H), 127.2 (*C*H), 126.7 (*C*H), 125.6 (*C*H), 110.3 (*C*H), 109.5 (*C*H), 48.4 (CH_2), 46.9 (CH_2) ppm (two isomeric sets of signals could be found in the ^1H and ^{13}C NMR spectra of **11c** relating to either the C2 (major signal) or C3 (minor signal) connected thiophene substituent (as no effect on the $^{11}B\{^1H\}$ NMR signal and the GPC trace could be observed) Whether this rearrangement is caused by photoirradiation *via* light or the interaction with the Ti catalyst during the dehydropolymerisation reaction could not be verified);[10]
$^{11}B\{^1H\}$ NMR (96 MHz, CD_2Cl_2, 22 °C) δ = -8.0 (br) ppm; GPC: M_n = 95,600 g mol^{-1}, PDI = 1.29.

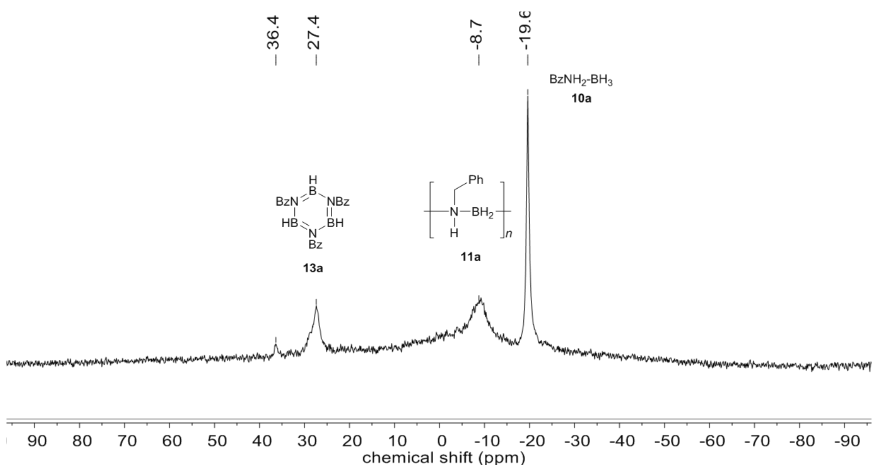

Figure S21: $^{11}B\{^1H\}$ NMR spectrum of **11a** in THF-d_8 (after 72 000 scans; weak polymer signal was detected due to partial solubility compared to the impurities of the sample).

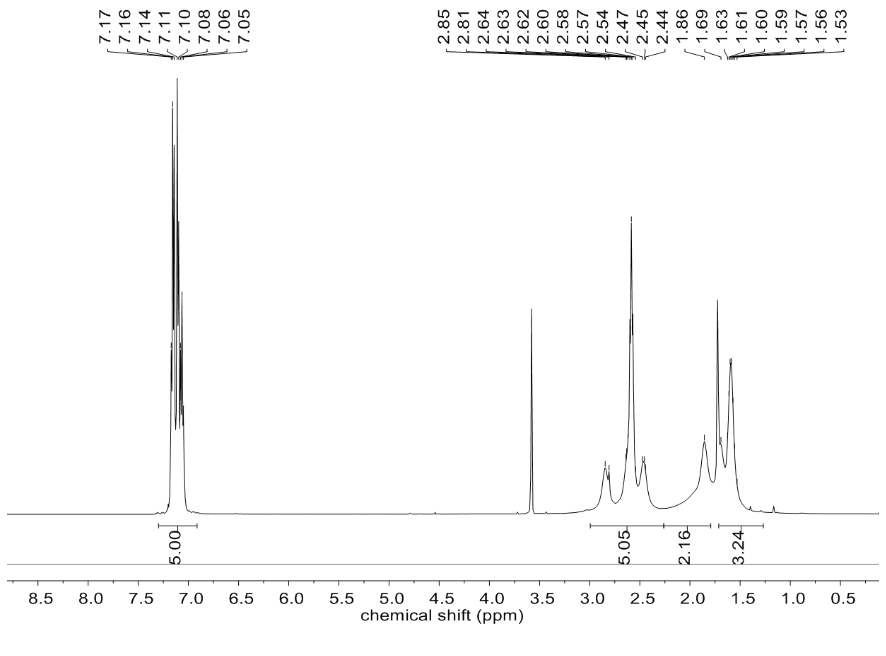

Figure S22: ^1H NMR spectrum of **11b** in THF-d_8.

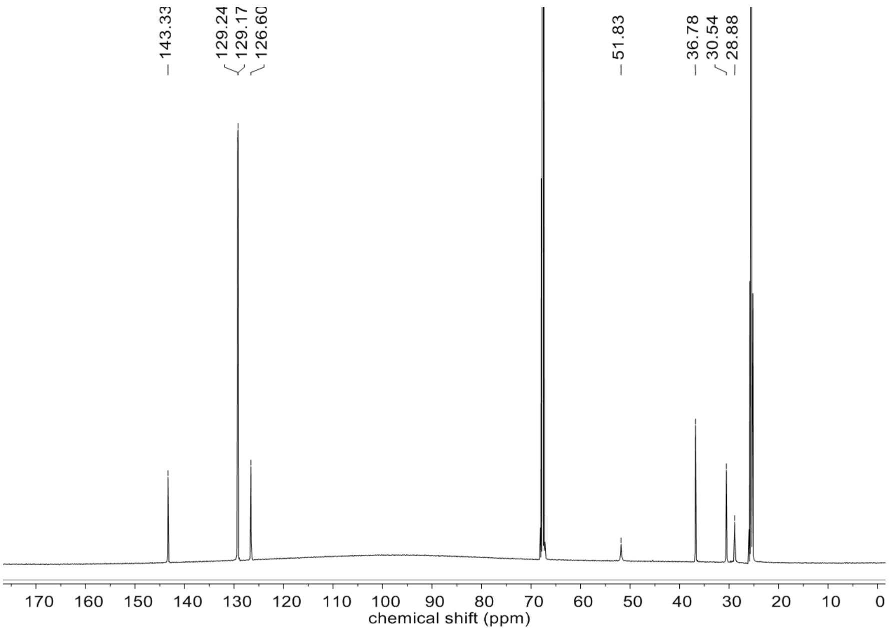

Figure S23: ^{13}C NMR spectrum of **11b** in THF-d_8.

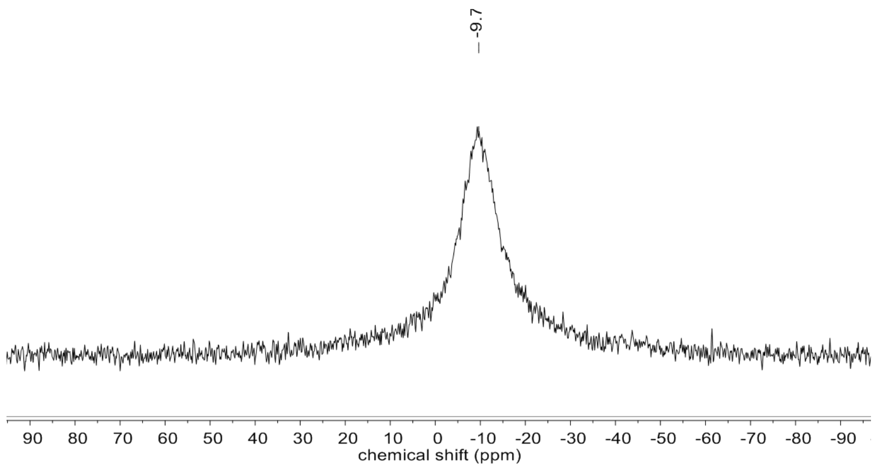

Figure S24: ^{11}B{^{1}H} NMR spectrum of **11b** in THF-d_8.

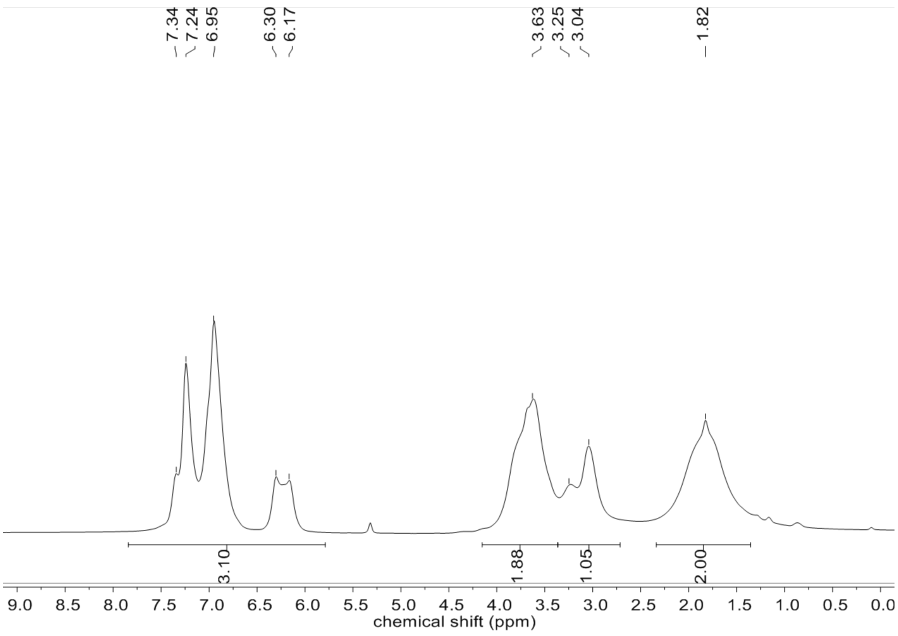

Figure S25: ^{1}H NMR spectrum of **11c** in CD_2Cl_2.

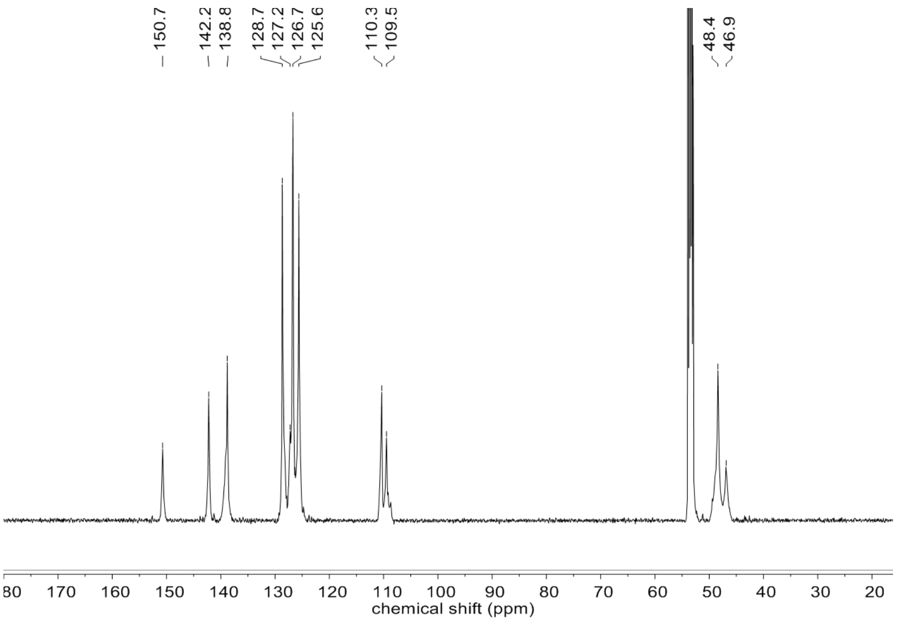

Figure S26: ^{13}C NMR spectrum of **11c** in CD_2Cl_2.

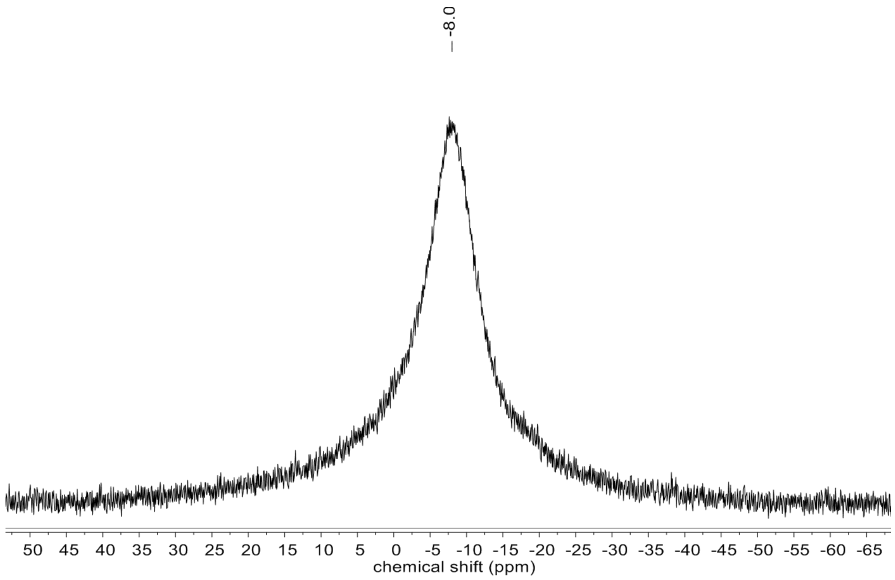

Figure S27: $^{11}B\{^1H\}$ NMR spectrum of **11c** in CD_2Cl_2.

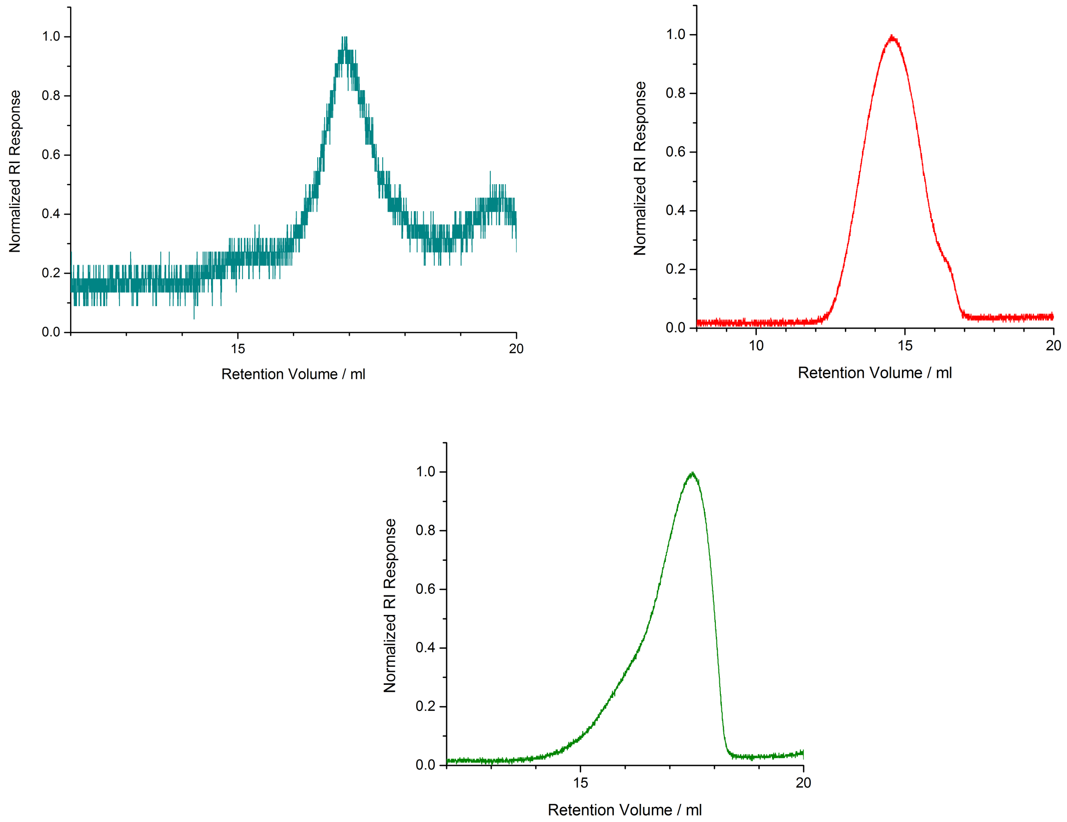

Figure S28: GPC traces for **11a** (*blue, top left*), **11b** (*red, top right*) and **11c** (*green, bottom*) from the reactions of **10a-c** with precatalyst [**6e** + 2*n*BuLi] (7 mol%, 8 h, 22 °C). See page S2 for more information.

Catalytic dehydropolymerisation of $BzNH_2{\cdot}BH_3$ (10a) and nBuNH$_2{\cdot}$BH$_3$ by precatalyst 6e (*optimised condition, 7 mol%, 8 h*) yielding copolymer 11d. To a solution of substrate **10a** (61 mg, 0.5 mmol) and nBuNH$_2{\cdot}$BH$_3$ **(10d)** (44 mg 0.5 mmol) in 1 mL of anhydrous toluene was added a solution of precatalyst [**6e** + 2nBuLi] (0.07 mmol) in 1 mL toluene. The solution was stirred at 22 °C for 8 h while the colour gradually changed from a dark green to a dark indigo/brown. Upon completion the reaction solution was transferred dropwise to vigorously stirred n-hexanes at room temperature and a white solid precipitated. The solid was filtered off and dried under vacuum (yield **11d**: 44%). The content of the resulting polymer was determined by integration of the ^{1}H NMR spectrum giving [BzNH-BH$_2$]$_n$-*r*-[nBuNH-BH$_2$]$_m$ n:m ≈ 2:1. Analysis for **11d**: ^{1}H NMR (500 MHz, THF-d_8, 22 °C) δ = 7.30 (br, 5 *H* CH_{aryl}), 4.25–1.25 (10.6 *H*, overlapping signals including: CH_2, N*H*, B*H*, THF), 1.25–0.50 (br, 1.5 *H*, CH_3) ppm; ^{13}C NMR (125.8 MHz, THF-d_8, 22 °C) δ = 137.5, 129.7, 128.0, 127.1, 54.2, 51.9, 20.9, 11.1 ppm; ^{11}B{^{1}H} NMR (96 MHz, THF-d_8, 22 °C) δ = -10 (br) ppm; GPC: M_n = 131 900 g mol^{-1}, PDI = 1.33.

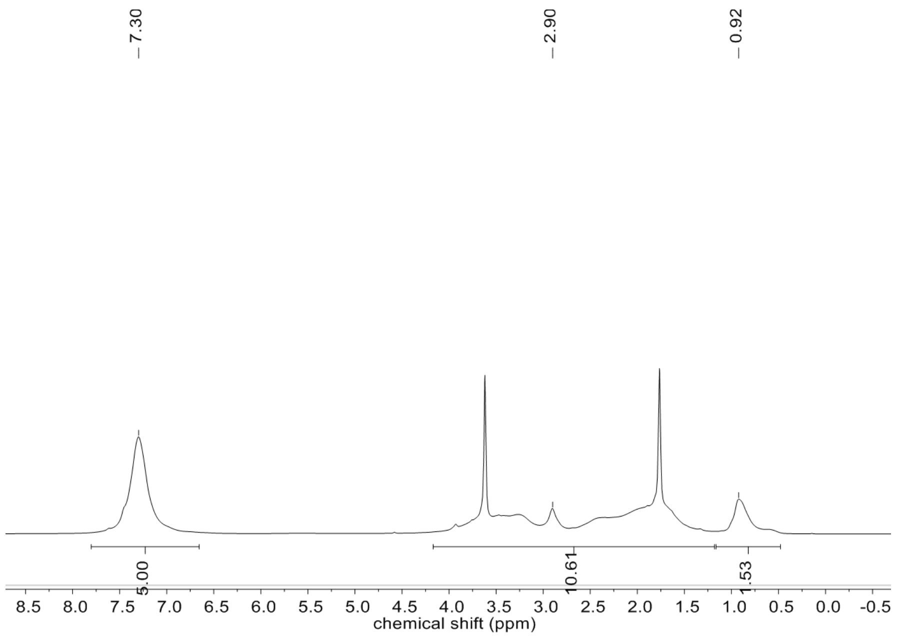

Figure S29: ^{1}H NMR spectrum of **11d** in THF-d_8.

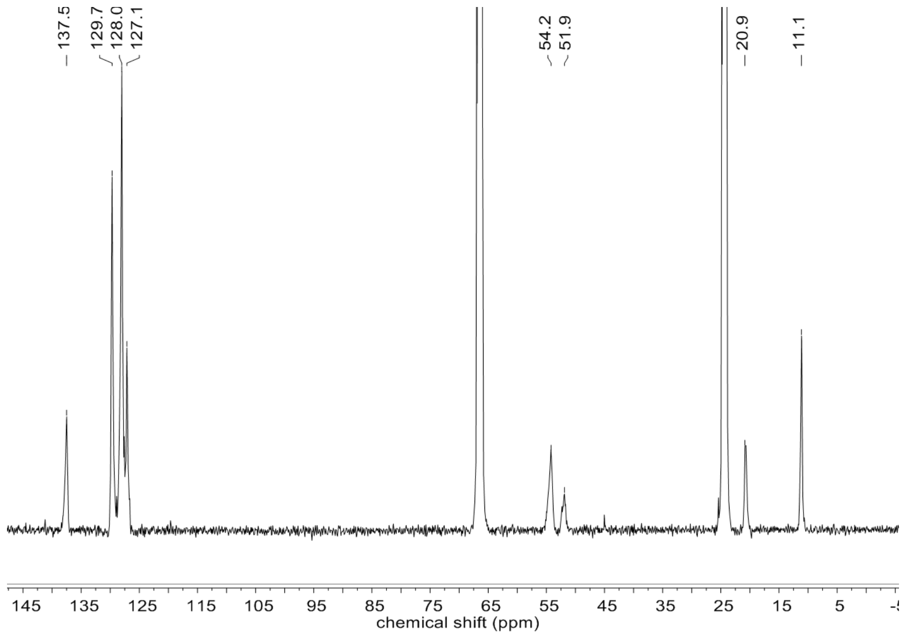

Figure S30: ^{13}C NMR spectrum of **11d** in THF-d_8.

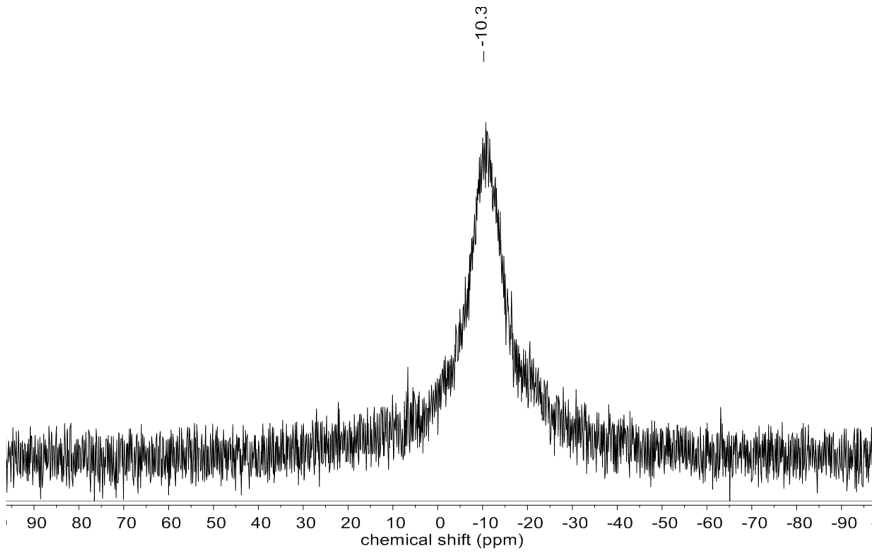

Figure S31: ^{11}B{^{1}H} NMR spectrum of **11d** in THF-d_8.

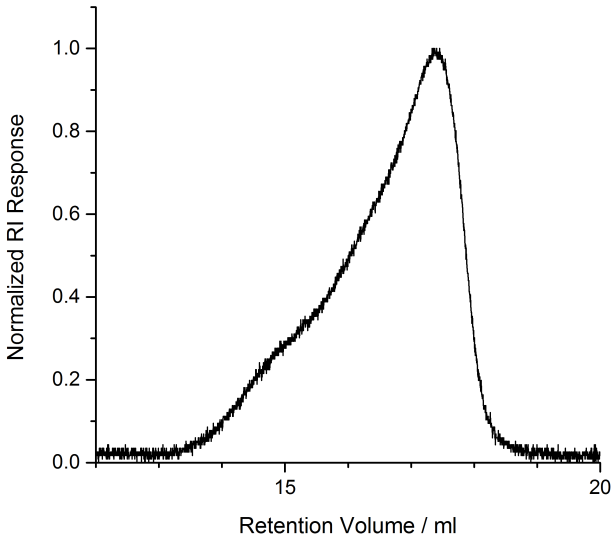

Figure S32: GPC traces for copolymer **11d** from the reactions of **10a** and [nBuNH$_2$·BH$_3$] with precatalyst [**6e** + 2nBuLi] (7 mol%, 8 h, 22 °C). See page S2 for more information.

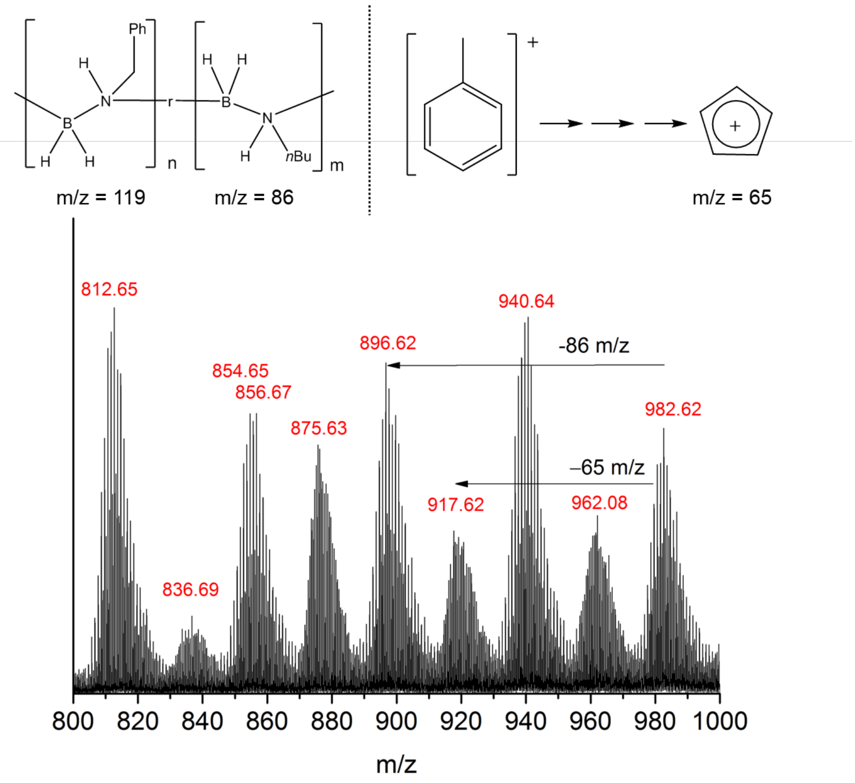

Figure S33: Expansion of ESI MS spectrum of [BzNH-BH$_2$]$_n$-r-[nBuNH-BH$_2$]$_m$ (**11d**).

Catalytic dehydropolymerisation of $MeNH_2 \cdot BH_3$ (4) by precatalyst 6e (*variable reaction time*). To a solution of substrate **4** (135 mg, 3.0 mmol), in 1 mL of anhydrous toluene was added a solution of precatalyst [**6e** + 2*n*BuLi] (0.21 mmol) in 1 mL toluene. The solution was allowed to stir at 22 °C for 0.5, 1, 2 or 4 h. The colour gradually changed from a dark green to a dark indigo/brown, and formation of gas was immediately noticeable. Upon completion, a 0.5 mL aliquot was taken and measured by $^{11}B\{^1H\}$ NMR (96 MHz) spectroscopy (Figures S19-S22). The formation of **5** ($\delta^{11}B$ -6.1 ppm) was evident from the broad peak at *ca.* $\delta^{11}B$ -18 ppm, which had previously been assigned to the polymer end-group $[MeNH\text{-}BH_2]_n\text{-}NMeH\text{-}BH_3$. The presence of unreacted **4** adding to the intensity of the resonance, however, cannot be excluded. All other peaks matched the expected shifts previously observed in the NMR test trials (Figure S8 and S9: **7** $\delta^{11}B$ -5.8 ppm, **8** $\delta^{11}B$ 32.5 ppm, and **9** $\delta^{11}B$ 27.7 ppm). The remaining reaction solution was transferred dropwise to vigorously stirred *n*-hexanes at -40 °C, and a white solid precipitated. The solid was filtered off and dried under vacuum (yield 62-74%). Resulting molecular weights were determined by GPC analysis (Figure S38) and are summarised in Table 2.

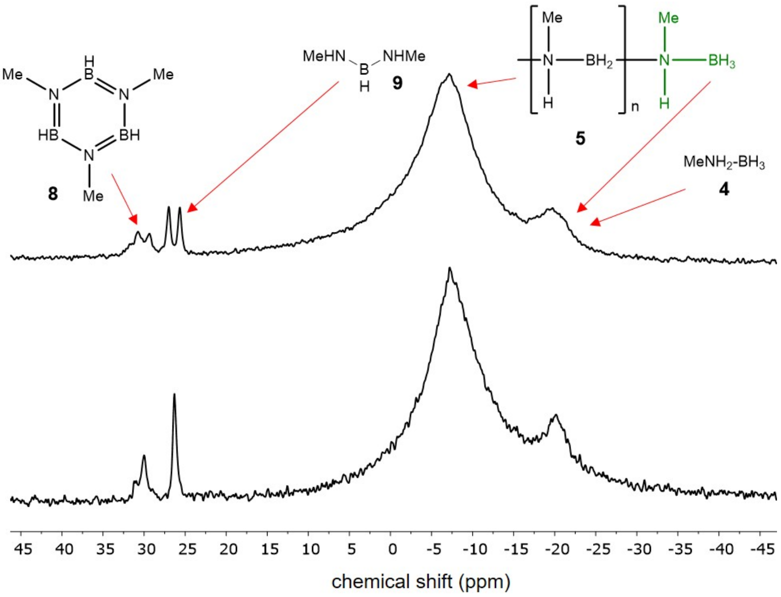

Figure S34: (*bottom*) $^{11}B\{^1H\}$ (96 MHz, toluene), (*top*) ^{11}B (96 MHz, toluene) NMR spectra of the reaction of **4** with precatalyst [**6e** + 2*n*BuLi] (7 mol%, 0.5 h, 22 °C).

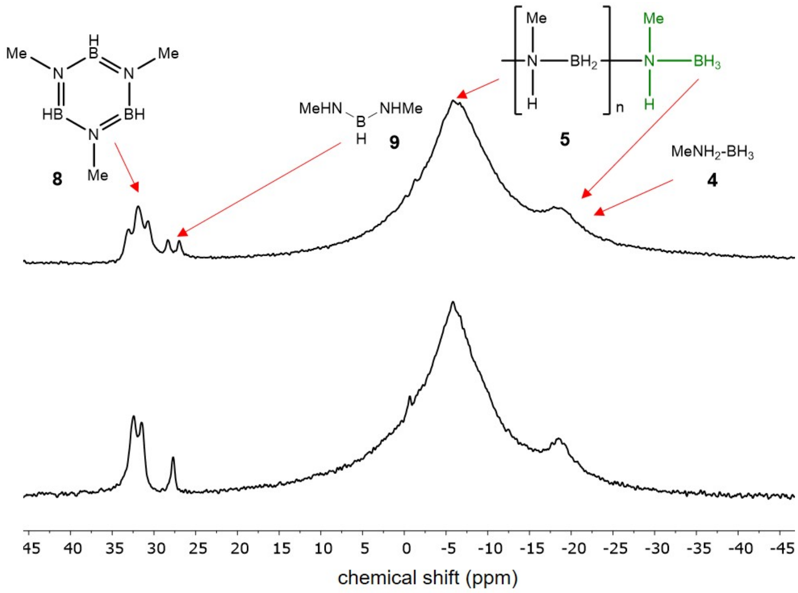

Figure S35: (*bottom*) $^{11}B\{^1H\}$ (96 MHz, toluene), (*top*) ^{11}B (96 MHz, toluene) NMR spectra of the reaction of **4** with precatalyst [**6e** + 2*n*BuLi] (7 mol%, 1 h, 22 °C).

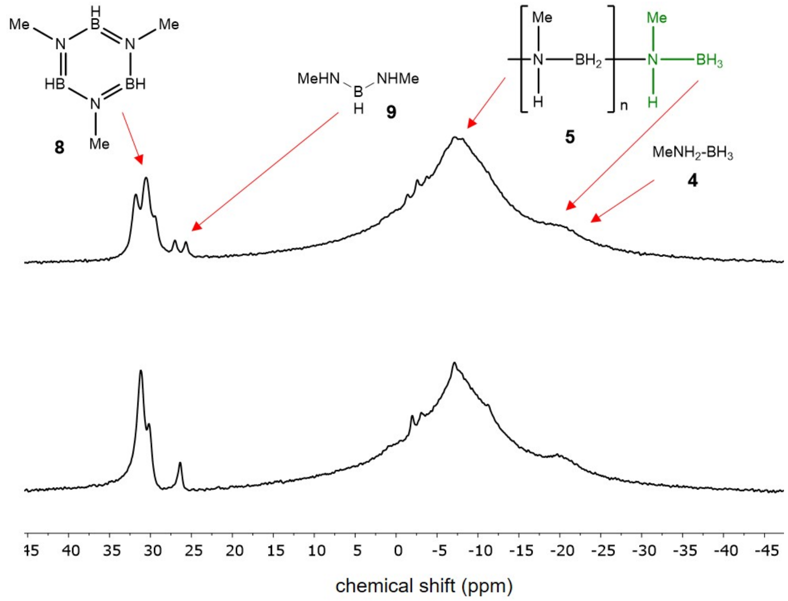

Figure S36: (*bottom*) $^{11}B\{^1H\}$ (96 MHz, toluene), (*top*) ^{11}B (96 MHz, toluene) NMR spectra of the reaction of **4** with precatalyst [**6e** + 2*n*BuLi] (7 mol%, 2 h, 22 °C).

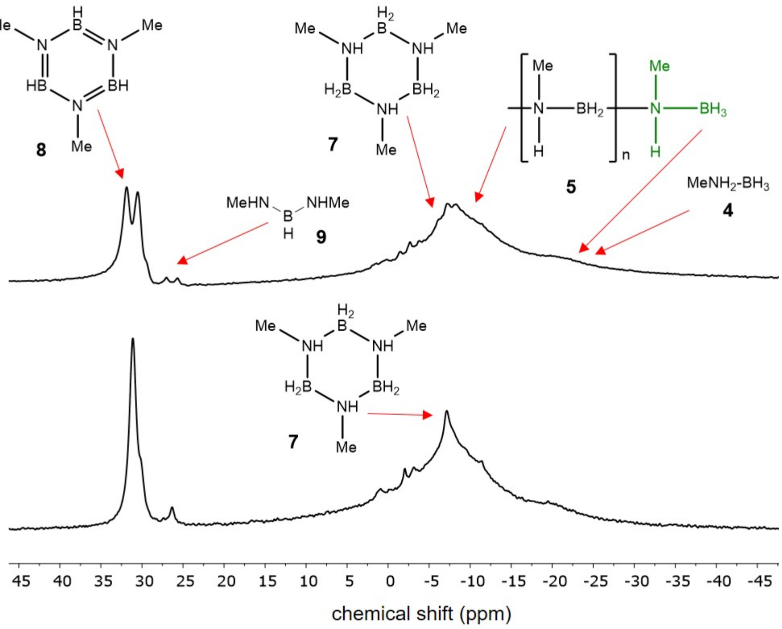

Figure S37: (*bottom*) $^{11}B\{^{1}H\}$ (96 MHz, toluene), (*top*) ^{11}B (96 MHz, toluene) NMR spectra of the reaction of **4** with precatalyst [**6e** + 2*n*BuLi] (7 mol%, 4 h, 22 °C).

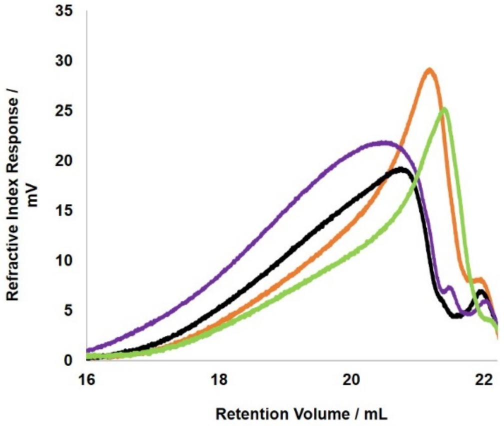

Figure S38: GPC traces for **5** from the reactions of **4** with precatalyst [**6e** + 2*n*BuLi] (7 mol%, **0.5 h**, **1 h**, **2 h**, and **4 h**, 22 °C). See page S2 for more information.

Depolymerisation [MeNH-BH$_2$]$_n$ (5) by precatalyst 6e (*NMR experiment*). To a solution of **5** (45 mg, M_n = 54 000 g mol^{-1}, and PDI = 1.32 by GPC), synthesised by literature methods with [IrH$_2$(POCOP)] (POCOP = 2,6-bis(di-tertbutylphosphinito)benzene) as catalyst,[3] in 0.9 mL of anhydrous toluene was added a solution of precatalyst [**6e** + 2*n*BuLi] (0.02 mmol) in 0.1 mL toluene (equivalent to 2 mol% catalyst). A 0.5 mL aliquot of the solution was then charged into a quartz J Young NMR tube with approximately 1.7 mL of headspace, then sealed and allowed to react at 22 °C. Solutions gradually changed from a dark green to a dark indigo/brown colour, and formation of gas was immediately noticeable. The reaction was monitored by ^{11}B{^{1}H} NMR (96 MHz) spectroscopy. Spectra were acquired at 5 min intervals for the first 60 min, followed by acquisitions at 30 min intervals for a total of 720 min (12 h), with additional measurements at 24 h, 48 h, and 176 h. Consumption of **5** (δ^{11}B -6.1 ppm) and concomitant formation of **8** (δ^{11}B 32.5 ppm), and a small amount of **9** (δ^{11}B 27.7 ppm) was immediately evident. After 176 h, all of **5** was consumed leaving only **8** as the final product (Figure S40).

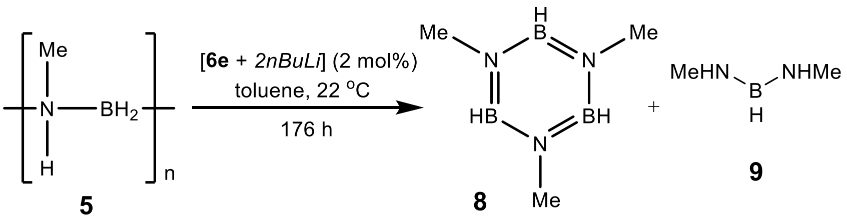

Scheme S1. Depolymerisation of **5** to products **8** and **9** utilizing precatalyst [**6e** + 2*n*BuLi] (2 mol%, 176 h, 22 °C).

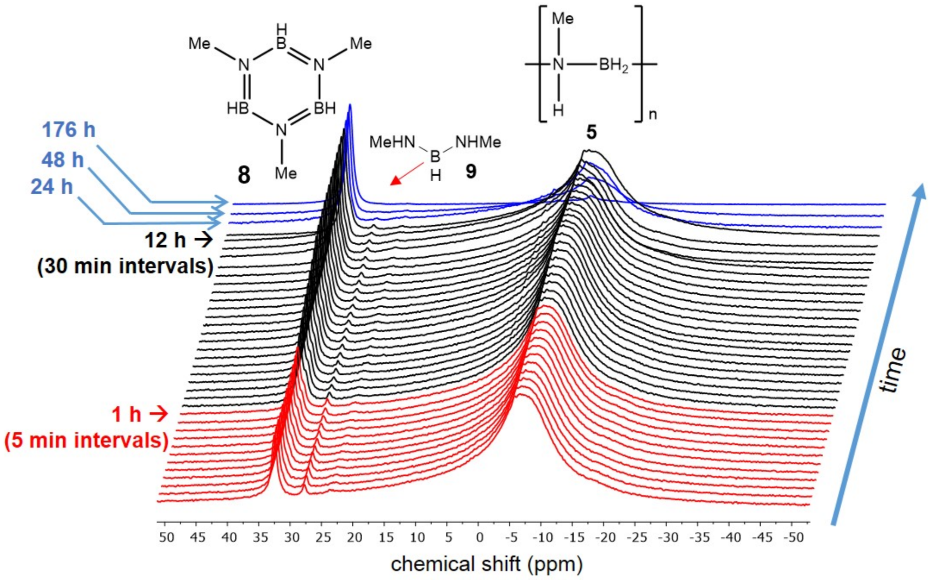

Figure S39: ^{11}B{^{1}H} (96 MHz, toluene) NMR spectra showing the depolymerisation of **5** to products **8** and **9** utilizing precatalyst [**6e** + 2*n*BuLi] (2 mol%, 176 h, 22 °C).

Catalytic dehydrogenation of [MeNH-BH$_2$]$_3$ (7) by precatalyst 6e (*NMR experiment*). To a solution of very pure (>99% by NMR spectroscopy) cyclotriborazane **7** (40 mg, 0.3 mmol) in 0.25 mL of anhydrous toluene was added a solution of precatalyst [**6e** + 2*n*BuLi] (0.02 mmol) in 0.25 mL toluene. The solution was allowed to react at 22 °C for 1 h, and was monitored by $^{11}B\{^1H\}$ NMR (96 MHz) spectroscopy (Figure S41). In this time, **7** was quantitatively dehydrogenated to borazine **8** ($\delta^{11}B$ 32.5 ppm, J_{BH} = 135 Hz), and no other byproducts were detected.

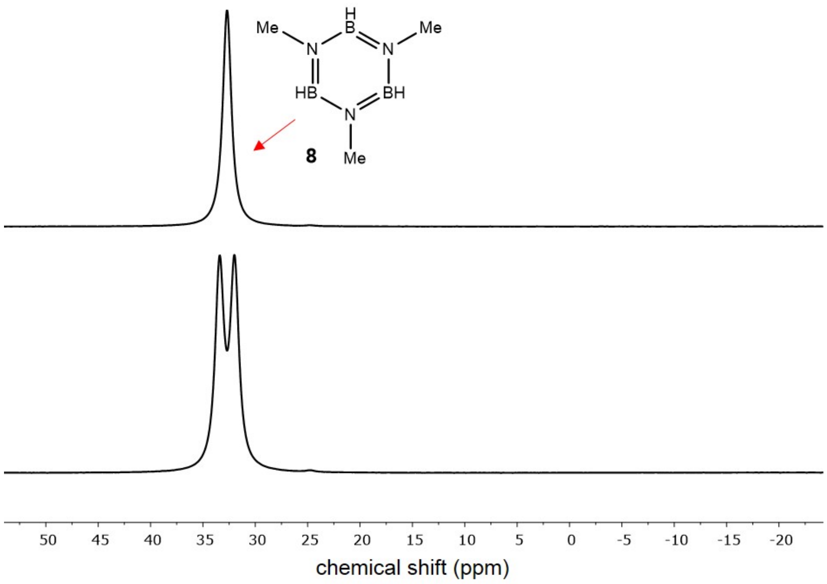

Figure S40: (*bottom*) ^{11}B (96 MHz, toluene), (*top*) $^{11}B\{^1H\}$ (96 MHz, toluene) NMR spectra showing the dehydrogenation of **7** (>99% by NMR) to **8** utilizing precatalyst [**6e** + 2*n*BuLi] (7 mol%, 1 h, 22 °C).

Cyclohexene trapping experiments for the catalytic dehydropolymerisation of MeNH$_2$·BH$_3$ (4) by precatalyst 6e. To a solution of substrate **4** (102 mg, 2.3 mmol), in 1.1 mL of anhydrous toluene was added a solution of precatalyst [**6e** + 2*n*BuLi] (0.05 mmol) in 0.75 mL toluene. Three 0.5 mL aliquots of the resulting solution were then charged into quartz J Young NMR tubes with approximately 1.7 mL of headspace. Five equivalents of cyclohexene were added 15 min after initiation of the reaction to test for the presence of free MeNH=BH$_2$ in solution. There was no difference in appearance (Figure S42, left) to that observed in the absence of cyclohexene. The NMR tube was then sealed and kept at 22 °C. The reaction was monitored by $^{11}B\{^1H\}$ NMR spectroscopy for the formation of the hydroboration product MeNH=BCy$_2$ ($\delta^{11}B$ 45 ppm) and polyaminoborane **5** ($\delta^{11}B$ -6.1 ppm). The addition of cyclohexene, however, had no effect on the catalyst activity and product distribution with polyaminoborane **5** ($\delta^{11}B$ -6.1 ppm) and varying amounts of by-products [MeNH-BH$_2$]$_3$, **7** ($\delta^{11}B$ -5.8 ppm), [MeN-BH]$_3$, **8** ($\delta^{11}B$ 32.5 ppm), and (MeNH)$_2$BH, **9** ($\delta^{11}B$ 27.7 ppm) being observed. (Figure S42, right). Nonetheless, the trapping experiment failed to yield the hydroboration product MeNH=BCy$_2$ (Figure S42, highlighted area marks the expected region for the hydroboration product[11]), either to the absence of free MeNH=BH$_2$ or the competing polymerisation reaction being significantly faster.

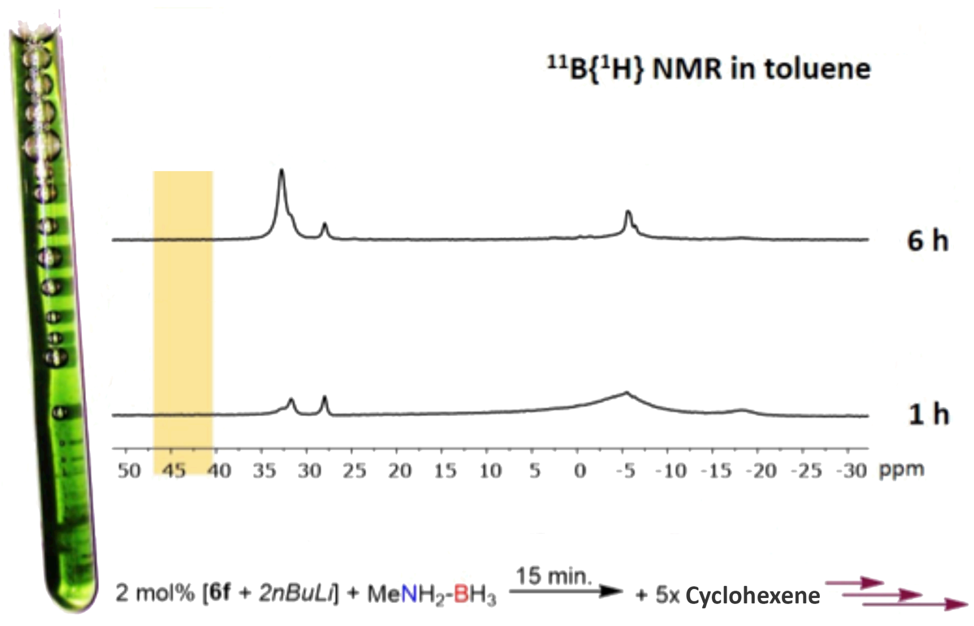

Figure S41: (*left*) photograph of the reaction mixture (*right*) $^{11}B\{^1H\}$ (96 MHz, toluene) NMR spectra of the cyclohexene trapping experiments (2 mol% **6e** + 2*n*BuLi, 6 h, 22 °C). Highlighted area marks the expected region for hydroboration product MeNH=BCy_2 (Cy = cyclohexyl).

Catalytic polycondensation of [MeNH-BH_2]$_x$ (5) by precatalyst 6e.

Part A (synthesis of 5): To a solution of **4** (135 mg, 3.0 mmol) in 1 mL of anhydrous toluene was added a solution of precatalyst [**6e** + 2*n*BuLi] (0.211 mmol) in 1 mL toluene. The solution was allowed to stir at 22 °C for 30 min. The deep blue colour of the catalyst solution remained throughout the experiment. The solution was then filtered into cold hexanes (-40 °C), upon which a colourless solid precipitated. This was isolated by centrifugation and dried *in vacuo* (yield: 84 mg). Analysis by $^{11}B\{^1H\}$ NMR spectroscopy indicated the presence of **8**, so the purification procedure was repeated (yield: 62 mg, 48%). The product was characterised by $^{11}B\{^1H\}$ NMR spectroscopy and GPC (M_n = 2,600 g mol^{-1}, PDI = 4.3) (Fig S42 and S43, red traces).

Part B (polycondensation of 5 from Part A): To a solution of **5** (55 mg, 1.2 mmol, ***from Part A***) in 1.8 mL of anhydrous toluene was added a solution of precatalyst [**6e** + 2*n*BuLi] (0.086 mmol) in 1.3 mL toluene. The solution was allowed to stir at 22 °C for 7.5 h. The deep blue colour of the catalyst solution remained throughout the experiment. The solution was then filtered into cold hexanes (-40 °C), upon which a colourless solid precipitated. This was isolated by centrifugation and dried *in vacuo* (yield: 16 mg, 30%). Analysis by $^{11}B\{^1H\}$ NMR spectroscopy indicated the presence of **5** and, potentially, a small amount of unreacted **4** as quantities did not permit further purification**.** The broad signal at $\delta^{11}B$ -18 ppm likely arises from the polymer end-group [MeNH-BH_2]$_n$-NMeH-*B*H_3, but in the absence of further purification (precluded here by the amount of material obtained) a contribution from unreacted **4**

excluded. The product was further characterised by GPC (M_n = 18,000 g mol^{-1}, PDI = 1.8) (Fig S42 and 43, blue traces).

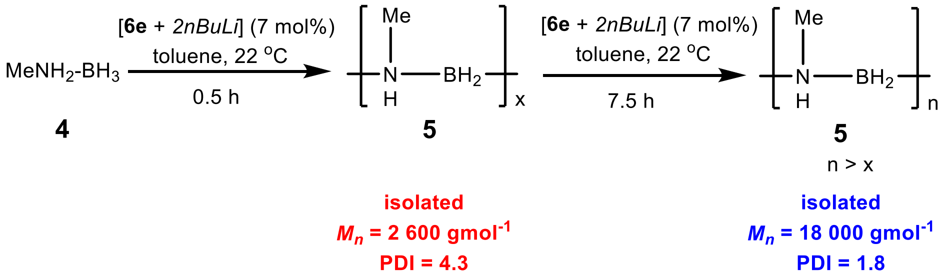

Scheme S2. Polymerisation of isolated **5** from the reaction of **4** with precatalyst **6e** + 2*n*BuLi (7 mol%, 0.5 h, 22 °C) with a second equivalent of precatalyst [**6e** + 2*n*BuLi] (7 mol%, 7.5 h, 22 °C).

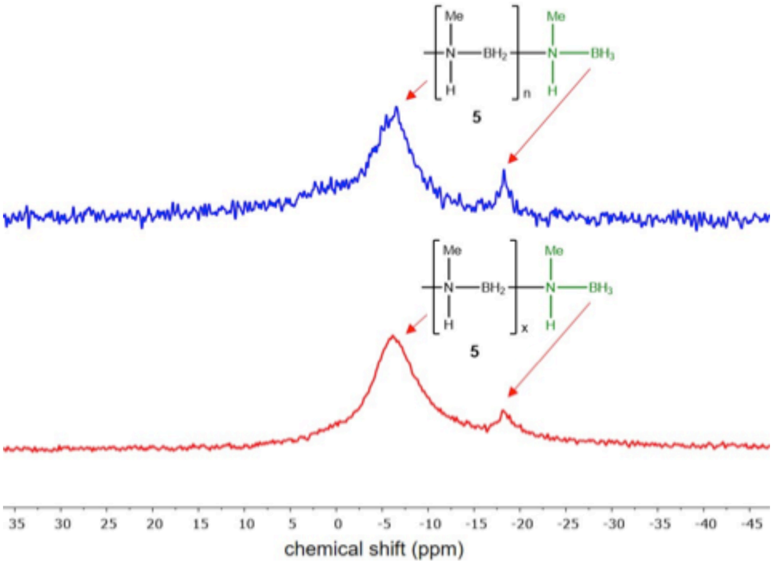

Figure S42: $^{11}B\{^1H\}$ (96 MHz, toluene) NMR spectra of (*bottom*) of low molar mass $[MeNH\text{-}BH_2]_x$ (**5**, M_n = 2,600 g mol^{-1}, PDI = 4.3) (from reaction of **4** + 7 mol% **6e** + 2*n*BuLi, 0.5 h, 22 °C) and (*top*) $[MeNH\text{-}BH_2]_n$ (**5**, M_n = 18,000 g mol^{-1}, PDI = 1.8) from the reaction of low molar mass $[MeNH\text{-}BH_2]_x$ (**5**) with a second equivalent of precatalyst [**6e** + 2*n*BuLi] (7 mol%, 7.5 h, 22 °C). *(**n** > **x**) Note: the broad signal at $\delta^{11}B$ -18 ppm is assigned to the polymer end-group of **5**, $[MeNH\text{-}BH_2]_n\text{-}NMeH\text{-}BH_3$, however, the presence of small amounts of unreacted **4** cannot be excluded.*

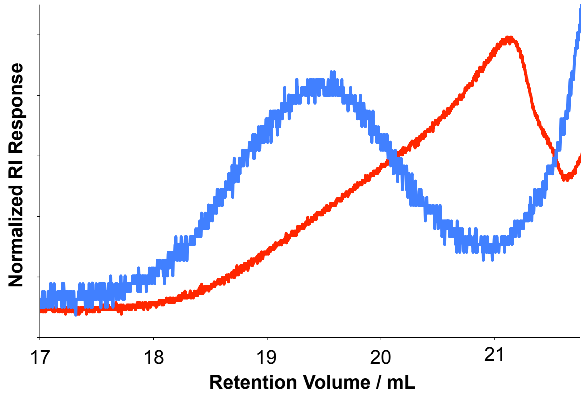

Figure S43: GPC traces of [MeNH-BH$_2$]$_x$ (**5**, M_n = 2,600 g mol^{-1}, PDI = 4.3) (from the reaction of **4** + 7 mol% **6e** + 2*n*BuLi, 0.5 h, 22 $^{\circ}$C) and [MeNH-BH$_2$]$_n$ (**5**, M_n = 18,000 g mol^{-1}, PDI = 1.8) isolated from the reaction of low molar mass [MeNH-BH$_2$]$_x$ (**5**) with a second equivalent of precatalyst [**6e** + 2*n*BuLi] (7 mol%, 7.5 h, 22 $^{\circ}$C) (***n*** > ***x***). See page S2 for further details.

Catalytic polycondensation of [MeNH-BH$_2$]$_x$ (5) by [IrH$_2$(POCOP)].

To a solution of **5** (M_n = 3,100 g mol^{-1}, PDI = 2.7) (30 mg, 0.668 mmol) in 0.35 mL of anhydrous THF at 0 $^{\circ}$C was added a solution of [IrH$_2$(POCOP)] (0.0068 mmol) in 0.35 mL THF (also at 0 $^{\circ}$C). The reaction solution was allowed to warm to 22 $^{\circ}$C then stirred for a further 20 minutes. The yellow solution was then filtered into cold hexanes (-40 $^{\circ}$C), upon which a pale yellow solid precipitated. This was isolated by centrifugation and dried in vacuo (yield: 0.033 g, >100%*). **Note: the high yield presumably results from the presence of residual catalyst in the sample, which would also account for the colour*. The product was characterised by ^{11}B{^{1}H} NMR spectroscopy and GPC (M_n = 6,700 g mol^{-1}, PDI = 2.5) (Fig S44 and S45).

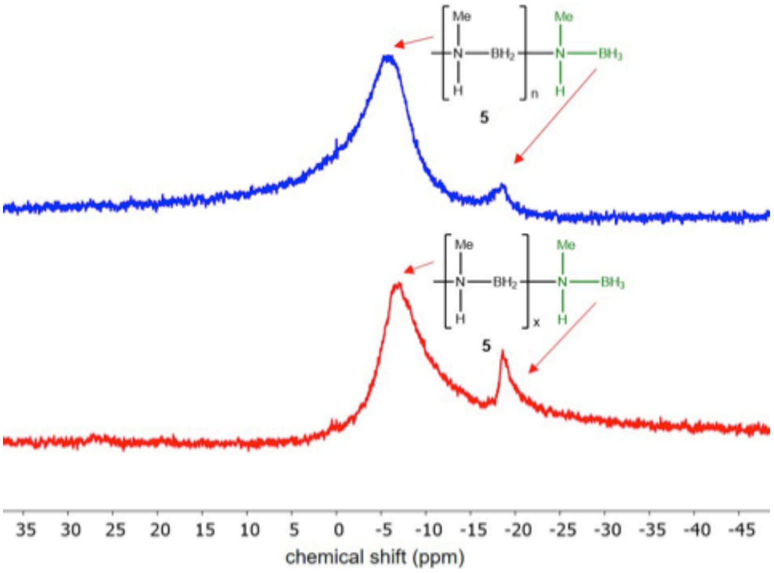

Figure S44: $^{11}B\{^{1}H\}$ (96 MHz, toluene) NMR spectra of (*bottom*) $[MeNH\text{-}BH_2]_x$ (**5**, M_n = 3,100 g mol^{-1}, PDI = 2.7) and (*top*) $[MeNH\text{-}BH_2]_n$ (**5**) isolated from reaction of $[MeNH\text{-}BH_2]_x$ (**5**) with 1 mol% $[IrH_2(POCOP)]$ (20 min, 0-22 $^{\circ}$C). ***(n > x)*** *Note: the broad signal at* $\delta^{11}B$ *-18 ppm is assigned to the polymer end-group of* ***5****,* $[MeNH\text{-}BH_2]_n\text{-}NMeH\text{-}BH_3$*, however, given the limited purification that was possible, the presence of compound* ***4*** *cannot be excluded.* See page S2 for further details.

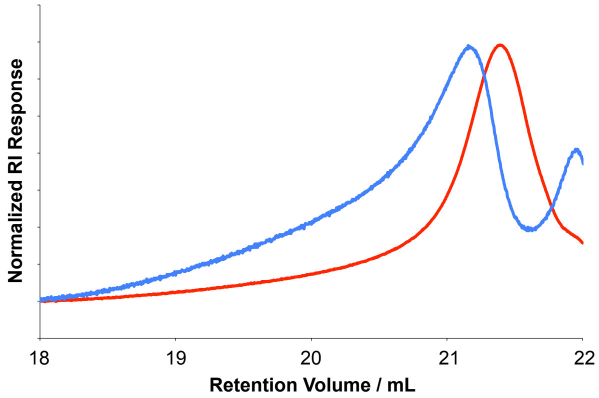

Figure S45: GPC chromatograms of $[MeNH\text{-}BH_2]_x$ (**5**, M_n = 3,100 g mol^{-1}, PDI = 2.7) and $[MeNH\text{-}BH_2]_n$ (**5**, M_n = 6,700 g mol^{-1}, PDI = 2.5) isolated from reaction of **5** $[MeNH\text{-}BH_2]_x$ with 1 mol% $[IrH_2(POCOP)]$ (20 min, 0-22 $^{\circ}$C) ***(n > x)***. See page S2 for further details.

Catalytic dehydropolymerisation of $MeNH_2{\cdot}BH_3$ (4) by $[IrH_2(POCOP)]$.

To a solution of $[IrH_2(POCOP)]$ (7 mg, 0.011 mmol) in 0.57 mL anhydrous THF at 0 $^{\circ}$C was added a solution of **4** (51 mg, 1.14 mmol), in 0.57 mL of anhydrous THF also at 0 $^{\circ}$C. The solution was allowed to warm to 22 ^{o}C then stirred for a further 20 min. The yellow solution was then filtered into cold hexanes (-40 $^{\circ}$C), upon which a pale yellow solid precipitated. The solid was isolated by centrifugation and dried

in vacuo (yield: 40 mg). The product was characterised by ^{11}B NMR spectroscopy and GPC (M_n = 262,600 g mol^{-1}, PDI = 1.7) (Figure S46 and S47).

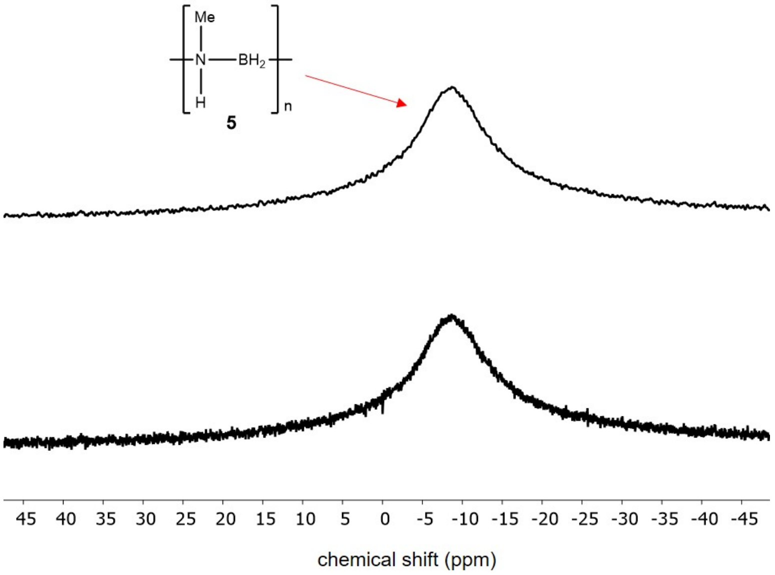

Figure S46: (*bottom*) $^{11}B\{^{1}H\}$ (96 MHz, toluene), (*top*) ^{11}B (96 MHz, toluene) NMR spectra of the reaction of **4** with 1 mol% [IrH_2(POCOP)] (20 min, 0-22 °C).

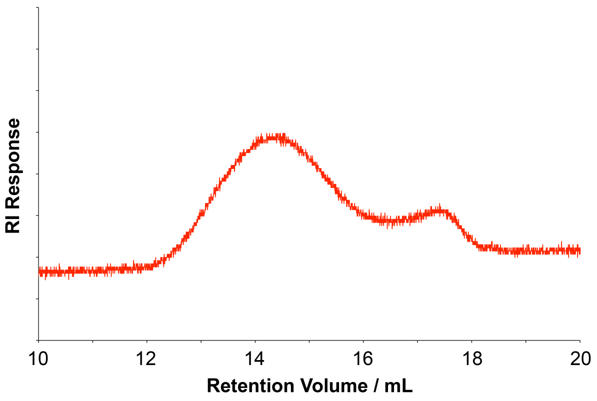

Figure S47: GPC trace of **5** (M_n = 262,600 g mol^{-1}, PDI = 1.7) isolated from reaction of **4** with 1 mol% [IrH_2(POCOP)] (20 min, 0-22 °C). See page S2 for further details.

References

[1] A. B. Pangborn, M. A. Giardello, R. H. Grubbs, R. K. Rosen and F. J. Timmers, *Organometallics*, 1996, **15**, 1518-1520.
[2] C. A. Jaska, K. Temple, A. J. Lough and I. Manners, *J. Am. Chem. Soc.* 2003, **125**, 9424-9434.
[3] A. Staubitz, A. Presa Soto and I. Manners, *Angew. Chem. Int. Ed.* 2008, **47**, 6212-6215.
[4] C. K. Narula, J. F. Janik, E. N. Duesler, R. T. Paine and R. Schaeffer, *Inorg. Chem.* 1986, **25**, 3346-3349.
[5] M. A. Peterson, A. Bowman and S. Morgan, *Synth. Commun.* 2006, **32**, 443-448.
[6] H. Helten, B. Dutta, J. R. Vance, M. E. Sloan, M. F. Haddow, S. Sproules, D. Collison, G. R. Whittell, G. C. Lloyd-Jones and I. Manners, *Angew. Chem. Int. Ed.* 2013, **52**, 437-440.
[7] J. A. Smith, J. Von Seyerl, G. Huttner and H. H. Brintzinger, *J. Organomet. Chem.* 1979, **173**, 175-185.
[8] M. E. Sloan, A. Staubitz, T. J. Clark, C. A. Russell, G. C. Lloyd-Jones and I. Manners, *J. Am. Chem. Soc.* 2010, **132**, 3831-3841.

[9] A. N. Marziale, A. Friedrich, I. Klopsch, M. Drees, V. R. Celinski, J. Schmedt auf der Günne and S. Schneider, *J. Am. Chem. Soc.* 2013, **135**, 13342-13355.
[10] H. Wynberg, R. M. Kellogg, H. van Driel and G. E. Beekhuis, *J. Am. Chem. Soc.* 1967, **89**, 3501-3505.
[11] A. P. M. Robertson, E. M. Leitao, and I. Manners, *J. Am. Chem. Soc.* 2011, **133**, 19322-19325: MeNH=BCy_2 observed at δ^{11}B 44.9 ppm in THF.
